# Learning Micro-C from Hi-C with diffusion models

**DOI:** 10.1371/journal.pcbi.1012136

**Published:** 2024-05-17

**Authors:** Tong Liu, Hao Zhu, Zheng Wang

**Affiliations:** Department of Computer Science, University of Miami, Coral Gables, Florida, United States of America; University of Michigan, UNITED STATES

## Abstract

In the last few years, Micro-C has shown itself as an improved alternative to Hi-C. It replaced the restriction enzymes in Hi-C assays with micrococcal nuclease (MNase), resulting in capturing nucleosome resolution chromatin interactions. The signal-to-noise improvement of Micro-C allows it to detect more chromatin loops than high-resolution Hi-C. However, compared with massive Hi-C datasets available in the literature, there are only a limited number of Micro-C datasets. To take full advantage of these Hi-C datasets, we present HiC2MicroC, a computational method learning and then predicting Micro-C from Hi-C based on the denoising diffusion probabilistic models (DDPM). We trained our DDPM and other regression models in human foreskin fibroblast (HFFc6) cell line and evaluated these methods in six different cell types at 5-kb and 1-kb resolution. Our evaluations demonstrate that both HiC2MicroC and regression methods can markedly improve Hi-C towards Micro-C, and our DDPM-based HiC2MicroC outperforms regression in various terms. First, HiC2MicroC successfully recovers most of the Micro-C loops even those not detected in Hi-C maps. Second, a majority of the HiC2MicroC-recovered loops anchor CTCF binding sites in a convergent orientation. Third, HiC2MicroC loops share genomic and epigenetic properties with Micro-C loops, including linking promoters and enhancers, and their anchors are enriched for structural proteins (CTCF and cohesin) and histone modifications. Lastly, we find our recovered loops are also consistent with the loops identified from promoter capture Micro-C (PCMicro-C) and Chromatin Interaction Analysis by Paired-End Tag Sequencing (ChIA-PET). Overall, HiC2MicroC is an effective tool for further studying Hi-C data with Micro-C as a template. HiC2MicroC is publicly available at https://github.com/zwang-bioinformatics/HiC2MicroC/.

## Author summary

The Micro-C assay, an improved variant of Hi-C, can capture genome-wide DNA interactions at nucleosome resolution, which makes it possible to detect more chromatin loops that relate to regulatory elements. However, there are not enough experimental Micro-C data publicly available compared with widely performed Hi-C experiments. Here we use deep learning methods to predict Micro-C from Hi-C. Our evaluation indicates that our method can successfully recover the loops that are detected in Micro-C but not identified in Hi-C. Our method provides an effective way to further take advantage of Hi-C.

## Introduction

The Hi-C techniques [1] have been extensively used to explore genome-wide chromatin three-dimensional (3D) structures. Studying Hi-C interaction maps results in finding varying levels of nuclear organization, including DNA loops [2,3], topologically associating domains (TADs) [4,5], and A/B compartments [1,2,6]. Hi-C data play a fundamental role in various areas, such as predicting DNA methylation [7,8], reconstructing 3D genome conformation [9,10], and studying the reprogramming of chromatin organization during mammalian embryogenesis [11,12]. However, restriction enzymes used in Hi-C experiments yield DNA fragments with ~ 4 kb in average length [13], which makes Hi-C cannot efficiently capture interactions associated with candidate cis-regulatory elements (cCREs) (range in size 150–350 base pairs [14]).

Micro-C [13,15], a Hi-C-based method, replaces restriction enzymes in Hi-C with micrococcal nuclease (MNase), which makes the captured interactions to single nucleosome (~100–200 base pairs) resolution. The Micro-C assay has been applied to different genomes and cell types, such as yeast [15] and mouse embryonic stem cells (mESCs) [16,17]. Most of the publicly available Micro-C data are for human cell types, including pluripotent human embryonic stem cells (H1-ESC) [18], differentiated human foreskin fibroblasts (HFFc6) [18], C42B [19], and K562 [20]. In multiple Micro-C studies [18,19,21], the same conclusion has been drawn, that is, we can identify significantly more loops with Micro-C than Hi-C. The massive increase in the number of identified loops is from the improvement of signal-to-noise ratio in Micro-C protocol, but not from the effect of increasing the number of valid read pairs [18]. Therefore, compared with Hi-C, Micro-C provides more multi-scale loops, enriching the interaction analysis between regulatory elements. However, compared with 8,268 Hi-C data sets in the NCBI GEO DataSets Database (as of Oct. 28, 2023), there are only 361 publicly available Micro-C results. The abundant Hi-C datasets can provide extra valuable looping interactions if we can effectively predict Micro-C from Hi-C.

The intuitive method to learn the mapping relationship from Hi-C to Micro-C is to perform regression analysis. Our previous works [22,23] have shown that deep neural networks can successfully enhance Hi-C resolutions by learning the regression relationships between down-sampled and high-resolution Hi-C contact matrices. Chromatin contact matrices can be predicted from one-dimensional (1D) factors, such as DNA sequence [24,25], epigenomic signals [26–28], and their hybrid [29]. Using 1D marks to predict 2D interactions usually results in generating massive false positive loops [30]. Based on the ChIP-seq matrix from the ENCODE project website, gathering various epigenomic marks for not-well-researched cell types as input is still a challenge. CAESAR [31] uses 1D and graph convolutional networks with low-resolution Hi-C and six epigenomic features as input to predict high-resolution Micro-C but needs 1D epigenomic tracks as features. The significant advantage of learning Micro-C from Hi-C is that we can improve the lower signal-to-noise in Hi-C without any other data type as input. Thus, we can detect more specific loops from predicted Micro-C, which usually cannot be identified in high-resolution Hi-C. In this way, we can make full use of high-resolution Hi-C to identify more significant loops.

Since the denoising diffusion probabilistic models (DDPM) [32,33] were introduced, they have been successfully modified and used for various tasks, such as text-conditional generative model DALL-E2 [34], time series imputation and forecasting [35], and image resolution enhancement [36]. Diffusion models also have been increasingly used in bioinformatics research [37], such as RFdiffusion [38] for generating protein structures and DiffDock [39] for modeling protein-ligand interaction. These studies [36,38] indicate that DDPM-based methods can perform better than vanilla regression ways even though the same deep networks are used.

In this work, we provide a DDPM-based computational method named HiC2MicroC to learn the mapping relationships between Hi-C and Micro-C. The only input of our method is Hi-C contact maps, which play a conditional role in DDPM. We trained our method using the Hi-C and Micro-C data from HFFc6 and evaluated our methods in six different cell types at two high resolutions (5 kb and 1 kb). Our evaluation results demonstrate that HiC2MicroC can successfully recover Micro-C loops that are not identified in Hi-C contact matrices. These recovered loops own similar genomic and epigenetic properties to real Micro-C loops.

## Material and methods

### Data for Hi-C and Micro-C

The Micro-C and Hi-C data for the two human cell types (H1-ESC and HFFc6) [18] were downloaded from the website of 4D Nucleome (S1 Table). The Hi-C data for H1-ESC have a relatively smaller number of valid pairs compared with the other three. The Hi-C, Micro-C, and promoter capture Micro-C (PCMicro-C) for C42B prostate cancer cells were obtained from gene expression omnibus (GEO) under the accession number GSE205000 [19]. The Micro-C data for K562 were downloaded from GEO, GSE206131 [20], and the raw Hi-C reads for K562 were obtained from GEO, GSE63525 [2], which were mapped to the reference genome (build hg38) [40] using Juicer (v1.9.9) [41].

We also benchmarked the methods in a mouse cell type. The Micro-C and Hi-C data for mESCs (S1 Table) are from [16] and [3], respectively. We downloaded the raw Hi-C reads of mESCs and mapped them to the reference genome (build mm10) [42] with Juicer. We randomly selected 2.6 billion valid read pairs from “merged_nondups.txt” with MAPQ ≥ 30, which were used to generate 5-kb and 1-kb contact matrices with cooler (v0.9.2) [43].

Finally, we use the Hi-C data of another well-studied human cell type GM12878, which does not have the corresponding high-resolution Micro-C data in the literature. The raw Hi-C reads of GM12878 were downloaded from GEO GSE63525 [2] and mapped to the reference genome (build hg38) using Juicer. The output file “merged_nodups.txt” was used to randomly extract 2.9 billion contact pairs with MAPQ ≥ 30, which were passed to “cload pairs” in cooler [43] to generate contact matrices at 5-kb and 1-kb resolution.

In total, we focus on six cell types in this paper, and the number of valid read pairs for both Hi-C and Micro-C of the six cell types varies from 400 million to 5.9 billion (S1 Table). It should be noted that we did not perform any sampling to make the six cell types have the same number of valid read pairs, which allows us to evaluate our method at different sequencing depths of Hi-C.

### Data processing and normalization

The large Hi-C and Micro-C contact matrices for each individual chromosome at 5-kb or 1-kb resolution are split into 256-by-256 submatrices. Considering the trade-off between the limitation of GPU memory and the training and prediction time, we choose 256-by-256 submatrices as our individual samples. Specifically, at 5-kb resolution we slide a window (256×256) with a 50-bin step in two directions (along the diagonal and towards the right). We only use the pixels within genomic distances less than 2 Mb, which is the same as the maximum loop length reported in Mustache. Therefore, the maximum number of steps towards the right is five. At 1-kb resolution, we change the step to 100, and the maximum number of steps towards the right is set to 20. The balanced values in each submatrix are extracted using the “fetch” function in cooler [43] and are further normalized to [–1, 1] by a four-step procedure: (1) values that are larger than a predefined “maxV” (0.05 for 5 kb and 0.08 for 1 kb) are set to “maxV”; (2) all values are linearly scaled to [1,10]; (3) a log10 transformation is applied to scale all values to [0, 1]; and (4) all values are linearly scaled to [–1, 1]. The “maxV” values are determined by choosing the approximate minimum element (excluding zero) of the diagonals of the two given offsets (0 and 1) from training data and are used to avoid skewness caused by the extremely larger pixels around the diagonals.

### Denoising diffusion probabilistic models

**Fig 1:**
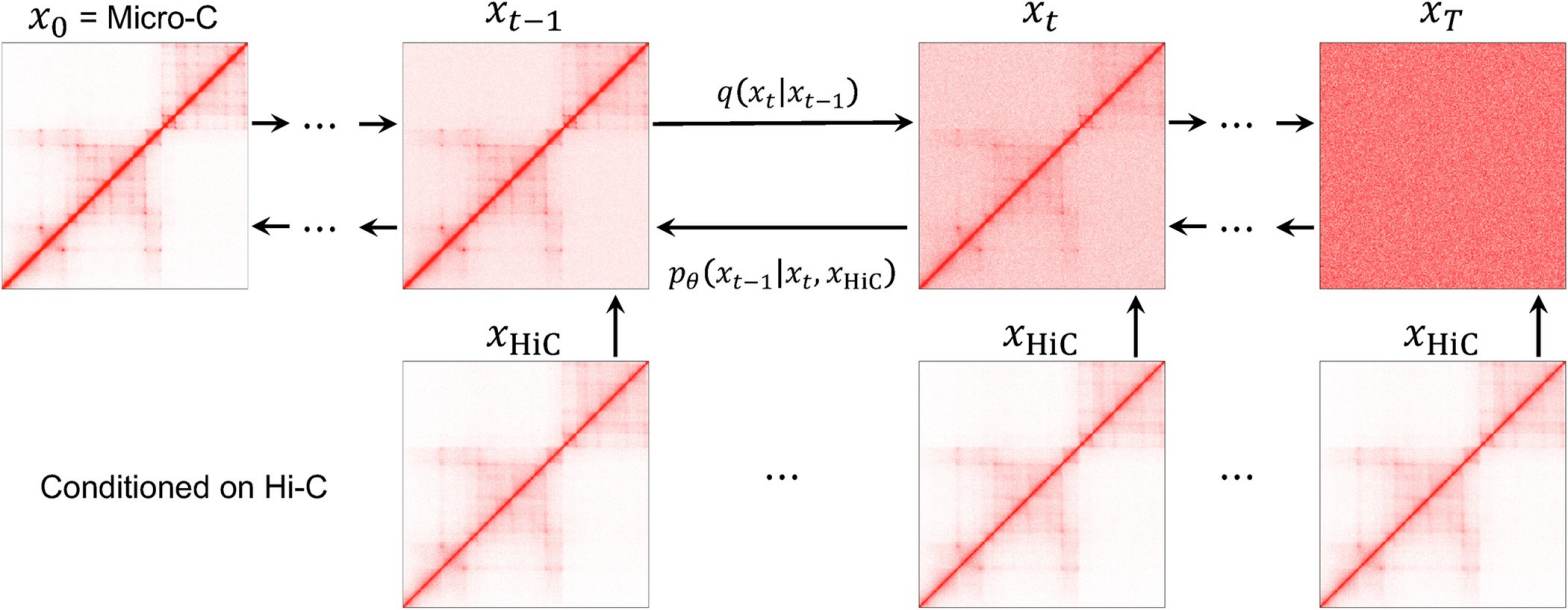
An illustration of DDPM used in HiC2MicroC. The forward diffusion process is shown as *q*(*x*_*t*_|*x*_*t*−1_). The learned reverse process *p_θ_*(*x*_*t*−1_|*x*_*t*_,*x*_HiC_) is conditioned on a Hi-C matrix.

The main part of HiC2MicroC is Hi-C-conditioned DDPM. In this section, we describe the procedures of the DPPM that we use. During training, DDPM has forward and reverse diffusion processes (Fig 1). The forward diffusion process gradually corrupts images *q*(*x*_*t*_|*x*_*t*−1_) by adding Gaussian noise to the previous noisy image (Fig 1) by $x_{t}=\sqrt{\beta_{t}}{\times \epsilon }_{t}+\sqrt{1-\beta_{t}}\times x_{t-1}$, where *ε*_*t*_∼*N*(0,*I*) at time step *t*, the linear noise schedule is set with the total time step *T* = 1000, and the variances are from *β*_1_ = 10^−4^ to *β*_*T*_ = 0.02. The noisy image *x*_*t*_ can directly be calculated from *x*_0_ (the uncorrupted Micro-C matrix) by $x_{t}=\sqrt{1-{\bar{\alpha }}_{t}}\times \epsilon +\sqrt{{\bar{\alpha }}_{t}}\times x_{0}$, where the cumulative product ${\bar{\alpha }}_{t}$ denotes *α*_1_*α*_*t*−1_⋯*α*_1_ and *α*_*t*_ = 1−*β*_*t*_. Therefore, during the forward diffusion process, we can directly obtain *x*_*t*_ from *x*_0_.

Starting from a random noisy image *x*_*T*_, the reverse diffusion process gradually denoises the image *p_θ_*(*x*_*t*−1_|*x*_*t*_,*x*_HiC_) (Fig 1) conditioned on the corresponding Hi-C matrix *x*_HiC_ using conditional distribution represented by deep networks (*θ* denotes parameters in neural networks). Since parameters of the deep network are shared across time steps, the sinusoidal position embedding borrowed from Transformers [44] is used to make deep networks know the exact time step (noise level).

The original DDPM simply uses the discrete index of the time steps as the input of embeddings [32], and the conditional generative model has the form *p_θ_*(*x*_*t*−1_|*x*_*t*_,*x*_HiC_,*t*). The major disadvantage of that is that the sampling/reference procedure is extremely time-consuming because we need to pass through each time step from *t* = 1000 to *t* = 0 to obtain the final sampled image *x*_0_. Therefore, we use the modified approach proposed by Wavegrad [45] that resolves this problem by replacing the discrete time step with a continuous noise level. Specifically, at time *t* we first calculate $l_{t}=\sqrt{\prod_{i=1}^{t}\left(1-\beta_{i}\right)}$ with *l*_0_ = 1, and then sample the new $\sqrt{{\bar{\alpha }}_{t}}$ from Uniform(*l*_*t*−1_,*l*_*t*_) (Algorithm 1). The new sampled $\sqrt{{\bar{\alpha }}_{t}}$ is used to obtain the corrupted image and to be the new continuous noise level. Therefore, the new distribution of the conditional generative model should be $p_{\theta }(x_{t-1}|x_{t},x_{HiC},\sqrt{{\bar{\alpha }}_{t}})$. During the inference/sampling procedure (Algorithm 2), we can set *T* to a small number (≪1000), which will significantly reduce the runtime without sacrificing the accuracy [45]. In total, we follow the original training and sampling procedures described in [32] with two major changes: (1) we add Hi-C matrices as a condition; (2) the new sampled $\sqrt{{\bar{\alpha }}_{t}}$ defined in Wavegrad is used to corrupt images and replace the noise level *t*.

**Algorithm 1** Training

1: **repeat**

2: *x*_0_ ~ *q*(*x*_0_)

3: *t* ~ Uniform (1, …, *T*)

4: $\sqrt{\bar{\alpha }}$ ~ Uniform (*l*_*t*-1_, *l*_*t*_)

5: ϵ∼N(0,I)

6: Take gradient descent step on

  $\nabla_{\theta }{\left\|\epsilon -\epsilon_{\theta }(\sqrt{\bar{\alpha }}x_{0}+\sqrt{1-\bar{\alpha }}\epsilon ,x_{HiC},\sqrt{\bar{\alpha }})\right\|}_{1}$

7: **until** converged

**Algorithm 2** Sampling (predicting)

1: *x*_*T*_ ~ N(0,I)

2: **for** *t* = *T*, …, 1 **do**

3:  *z* ~ N(0,I) if *t* > 1, else *z* = 0

4: $x_{t-1}=\frac{1}{\sqrt{\alpha_{t}}}\left(x_{t}-\frac{1-\alpha_{t}}{\sqrt{1-{\bar{\alpha }}_{t}}}\epsilon_{\theta }(x_{t},x_{HiC},\sqrt{{\bar{\alpha }}_{t}})\right)+\sigma_{t}z$

5: **end for**

6: **return** *x*_0_

We use the U-Net [46] architecture (S1A Fig) as our deep network, which contains two main stages (down-sampling and up-sampling). We follow the U-Net implementation in https://github.com/lucidrains/denoising-diffusion-pytorch. Both down-sampling and up-sampling stages contain residual blocks [47] and attention blocks [44]. In the residual block (S1B Fig), both scaling and shifting *x*×(1+*scale*)+*shift*) are used for integrating the embeddings of the noise level $\sqrt{{\bar{\alpha }}_{t}}$ into the network. The attention block (S1C Fig) includes a residual connection, a normalization layer, and an attention layer. Two attention mechanisms are used (vanilla [44] and linear attention [48]). Specifically, we use vanilla attention in the middle block of the U-Net and linear attention in the other blocks. All normalization layers are set to group normalization [49]. The number of groups in residual and attention blocks is four and one, respectively. The Sigmoid Linear Unit (SiLU) function [50] is used in residual blocks. For each down-sampling and up-sampling block in U-Net (S1D Fig), there are two residual blocks followed by a linear attention block and then a down-sampling/up-sampling operation.

### Regression models

We also trained regression models to compare with diffusion models. The regression models use the same U-Net as described in DDPM. The input and ground truth for the regression models are Hi-C and Micro-C, respectively. The regression model (U-Net) does not need noise level as input in the residual blocks and directly outputs the predicted Micro-C, whereas U-Net in DDPM outputs the predicted Gaussian noise.

### Training, validation, and blind test

In this study, we only use Hi-C and Micro-C from HFFc6 as training and validation data. The learned models are blindly tested on all the other cell types. For 5-kb resolution, we trained our DDPM and regression models with all chromosomes from HFFc6 excluding two chromosomes for validation (chromosome 17) and blind test (chromosome 1). For downstream analysis, the evaluation results at 5-kb resolution are based on chromosome 1 for all cell types. For 1-kb resolution, we left three chromosomes (1, 5, and 10) for blind test and the others were used for training.

### Implementation details

All Hi-C and Micro-C contact matrices are stored in the cool format designed in cooler [43]. If the 2D contact data we downloaded are in hic format, we used “hicConvertFormat” in HiCExplorer (v3.0) [51] to convert hic to cool. The balanced method implemented in cooler with default parameters is used to balance the raw contact matrices. After obtaining the predicted submatrices, we use the reverse version of the procedure described in “Data processing and normalization” to make the predicted Micro-C values have the real Micro-C scale. We then combine the submatrices into a large matrix for each testing chromosome. If a pixel is predicted more than one time, we use the average value as the final prediction. The large matrix is further converted into the cool format using the command “cooler load -f coo -count-as-float” in cooler.

We implemented our diffusion models in Pytorch (v1.12.1) [52] based on two DDPM implementations (https://github.com/lucidrains/denoising-diffusion-pytorch and https://github.com/lmnt-com/wavegrad). The loss function is fixed to L1 loss for both training and validation of DDPM and regression models. We used Adam [53] as the optimizer. For training 5-kb matrices, we initially set the learning rate to 1e-4 and reduce it by a factor 0.1 if the validation loss does not improve after 10 epochs. For training with 1-kb matrices, we fixed the learning rate to 1e-4 and trained DDPM and regression models for 30,000 steps. For both resolutions, we set the batch size to 16. We used the same hyperparameters for training the regression model as in DDPM. All models were trained on an NVIDIA A100 equipped with 40 GB memory. The depth multiplier is fixed to 1, 2, 4, and 8.

While sampling from the reverse process (Algorithm 2), we fixed *T* = 50 and *β*_1_ = 10^−4^ and tested different values for *β*_*T*_. The noise level $\sqrt{{\bar{\alpha }}_{t}}$ curves against time steps are shown in S2A Fig, demonstrating that the curve with larger *β*_*T*_ has a more similar shape to the original curve (*T* = 1000 and *β*_*T*_ = 0.02). Furthermore, the larger *β*_*T*_ corresponds to a smaller validation loss (S2B Fig). Therefore, we finally selected *β*_*T*_ = 0.95 for all our sampling procedures in this paper. The loss curves for training and validation of HiC2MicroC and regression are shown in S2C Fig.

### Evaluation metrics

The main evaluation method is to assess whether our recovered loops/peaks detected from our predicted Micro-C have similar properties to the loops identified from experimental Micro-C. We use two loop identification methods Mustache (v0.1.9) [21] and SIP (v1.6.1) [54]. Since SIP is not evaluated on 1-kb contact matrices by its authors [54], we only use Mustache to detect loops at 1-kb resolution. For running Mustache at 5-kb and 1-kb resolution, we test two false discovery rates (FDR) (0.05 and 0.1) and use sparsity thresholds 0.88 for 5 kb and 0.7 for 1 kb, which are default values in Mustache. For running SIP at 5-kb resolution, we also test two FDRs (0.01 and 0.05). For both Mustache and SIP, the other parameters are with default values.

We follow aggregate peak analysis (APA) described in [2] to averagely indicate the presence of loops. For performing APA on Hi-C and Micro-C data, we use observed/expected matrices calculated by cooltools (v0.5.4) [55], in which way we can use all loops without considering the effects of different genomic distances. For APA of PCMicro-C and Chromatin Interaction Analysis by Paired-End Tag Sequencing (ChIA-PET), we only use the loops within genomic distances from 100 kb to 2 Mb because of the unavailability of the observed/expected matrices. To perform APA at 5-kb resolution, we extract 11×11 submatrices centered at loop pixels and calculate APA scores for each of the four 3×3 corners (lower-left, lower-right, upper-left, and upper-right). To perform APA at 1-kb resolution, the shape of submatrices is changed to 51×51, and the corner size is increased to 15×15.

## 3 Results

### 3.1 Micro-C loops can be identified in HiC2MicroC-predicted matrices

#### 3.1.1 Loop counts and effectiveness of recovering Micro-C loops

**Fig 2:**
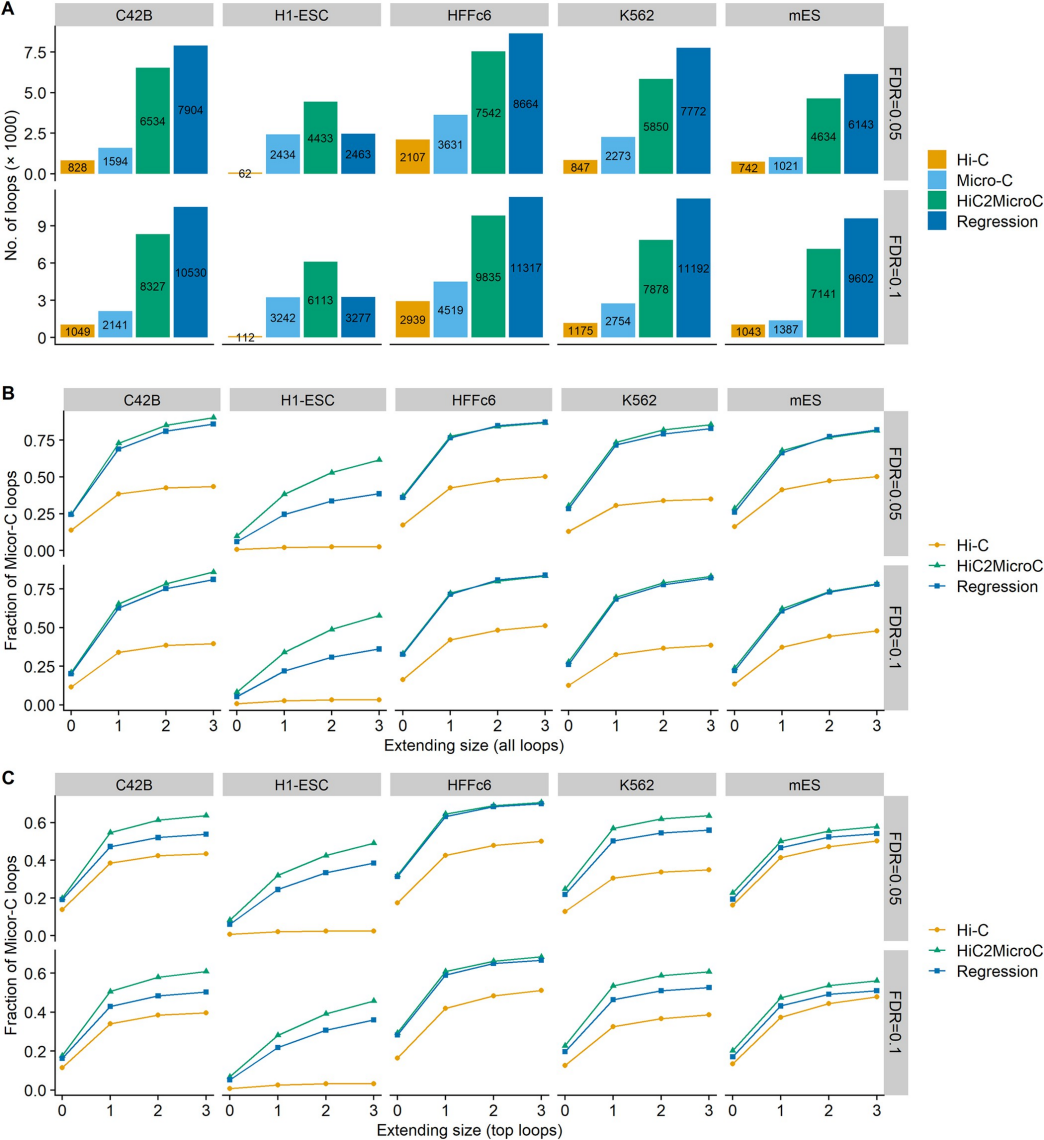
(A) The number of loops identified by Mustache on Hi-C, Micro-C, HiC2MicroC, and regression at 5-kb resolution. (B) The fraction of recovering Micro-C loops for all Mustache-detected loops from Hi-C, HiC2MicroC, and regression. (C) The same as (B) but on top loops.

The number of loops identified by Mustache and SIP at 5-kb resolution for five different cell types is shown in Figs 2A and S3A, respectively. When using Mustache with two FDRs (0.05 and 0.1), regression always results in more loops, followed by HiC2MicroC, Micro-C, and Hi-C (Fig 2A). An exception is H1-ESC in which HiC2MicroC obtains the most loops. When using SIP with two FDR (0.01 and 0.05), HiC2MicroC and regression identify a similar number of loops, followed by Micro-C and Hi-C. H1-ESC is still the exception (S3A Fig). We believe this is because the Hi-C matrices of H1-ESC are too sparse to identify a reasonable number of loops, which also affects the loops of HiC2MicroC and regression.

Next, we assess the fraction of reference (Micro-C) loops that HiC2MicroC and regression can recover (Figs 2B and S3B). The pixels surrounding a loop pixel are usually also enriched for contact frequency and detected as potential loops. Therefore, when examining whether a Micro-C loop is found in the other three loop sets, we set an extending size *s* (0, 1, 2, 3). When the extending size is equal to zero, the two loops are matched only if the indexes of the two corresponding pixels are the same. When the extending size is greater than zero, we think a Micro-C loop is recovered if there is at least one loop found in a (2*s*+1)×(2*s*+1) square centered at the loop pixel. For Mustache-detected loops (Fig 2B), we find that both HiC2MicroC and regression can recover more than 80% of the reference loops on five out of six cell types when the extending size is set to three. HiC2MicroC performs significantly better than regression in H1-ESC, and slightly better in C42B and K562. Both HiC2MicroC and regression apparently outperform Hi-C. For SIP-identified loops (S3B Fig), we can draw the similar conclusions.

One may argue that the improvement of recovering reference loops (compared to Hi-C) may come from the vast number of loops of HiC2MicroC and regression. Therefore, we further examine the fraction of Micro-C loops from top loops. The top loops for both HiC2MicroC and regression are those with the same number of Micro-C loops and having the smallest FDRs. Since the output loop files of SIP do not contain FDRs, we do not evaluate with respect to top loops from SIP. The larger fractions of recovering Micro-C loops from top loops (Fig 2C) indicate that HiC2MicroC still outperforms regression in almost all cell types, and both successfully recover more loops than Hi-C.

We report loop distance distribution in S4 Fig. For Mustache-detected loops, regression makes longer loops than the other three. For SIP-detected loops, the four methods have similar distribution. The results shown in S4 Fig also indicate that both HiC2MicroC and regression can generate short-range loops.

#### 3.1.2 Evaluation with APA

**Fig 3:**
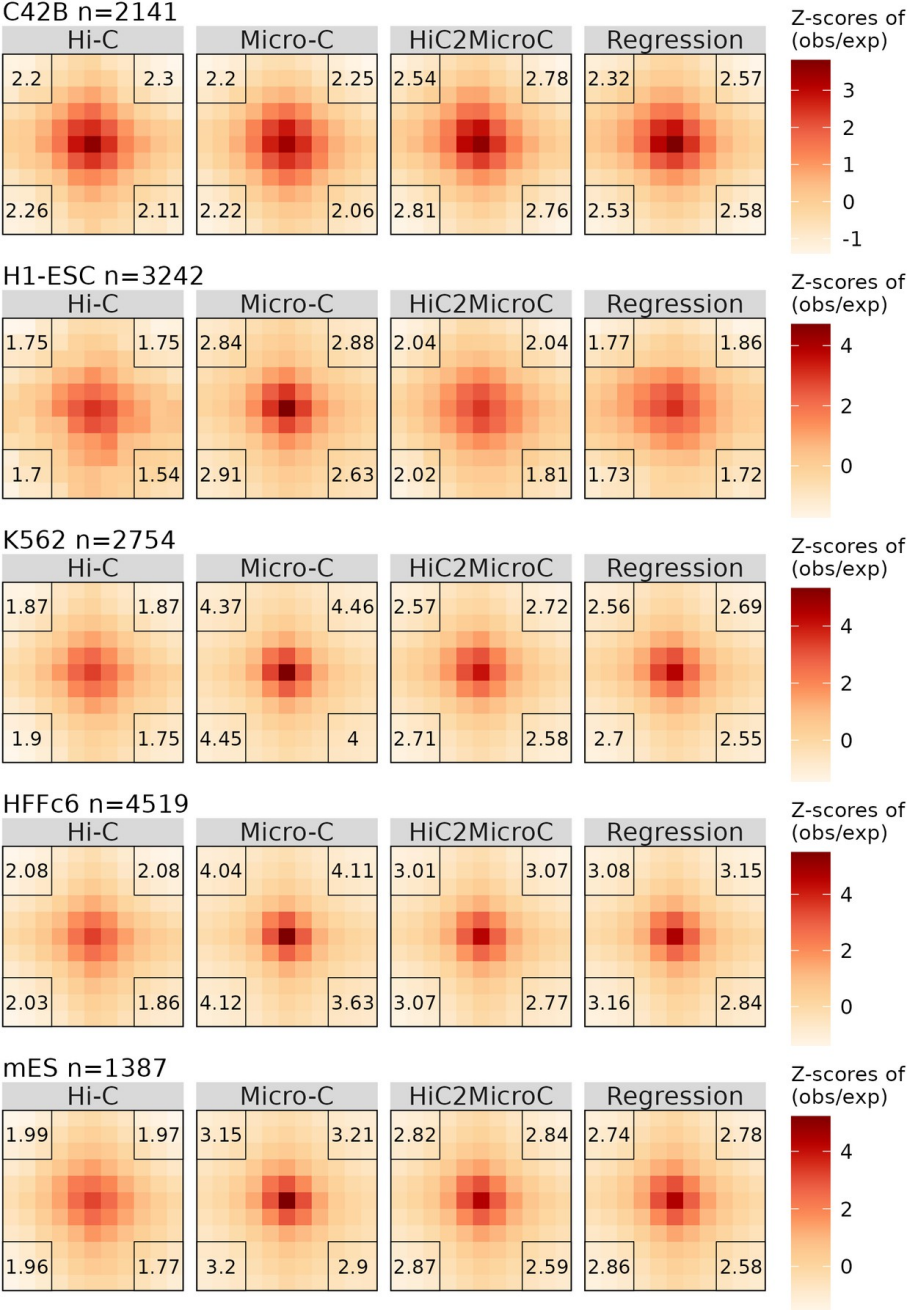
APA plots for all Mustache-detected Micro-C loops (FDR = 0.1) on matrices of Hi-C, Micro-C, HiC2MicroC, and regression in five cell types. The APA scores are shown at each corner. The number of loops used for generating APA plots is also provided beside cell type names.

We perform APA using Micro-C loops and the contact matrices of Hi-C, Micro-C, and predictions from HiC2MicroC and regression. We first examine all Micro-C loops on the four types of contact matrices. APA heat maps for all Mustache loops (FDR = 0.1 in Fig 3 and FDR = 0.05 in S5 Fig) and SIP loops (FDR = 0.05 in S6 Fig and FDR = 0.01 in S7 Fig) indicate that both HiC2MicroC and regression improve APA scores in almost all cell types. HiC2MicroC achieves higher APA scores than regression on Mustache-detected loops, whereas regression works better with SIP-detected loops.

Furthermore, we perform APA with Micro-C-specific loops (excluding Hi-C loops with the extending size set to two) on the four types of contact matrices. APA heat maps for Mustache-specific loops (FDR = 0.1 in S8 Fig and FDR = 0.05 in S9 Fig) and SIP-specific loops (FDR = 0.05 in S10 Fig and FDR = 0.01 in S11 Fig) also demonstrate that the predicted Micro-C from HiC2MicroC and regression correspond to higher APA scores than Hi-C with respect to Micro-C-specific loops, indicating that our methods reshape the original Hi-C and make it much closer to the corresponding Micro-C. For downstream analysis, we will mainly focus on the top Mustache-detected loops to lessen the effects of the different number of loops between Micro-C and our predictions.

#### 3.1.3 Ablation studies

In this subsection, we first explore the effect of different Hi-C sequencing depths as input, and then determine whether random sample (*x*_*T*_ in Algorithm 2) affect the robustness of HiC2MicroC. Finally, we try to prove noise level serves a crucial role during prediction.

The number of read pairs shown in S1 Table and loop numbers shown in Figs 2A and S3A demonstrate that HiC2MicroC is robust to different Hi-C sequencing depths (ranging from 0.4B to 2.9B), and more Hi-C reads result in more significant loops. We further reduced Hi-C reads by randomly down-sampling raw Hi-C read pairs with three ratios (1/4, 1/8, and 1/16) in HFFc6 and K562. The number of Mustache-detected (FDR = 0.05) loops with original and down-sampled Hi-C as input is shown in S12A Fig. Surprisingly, the number of loops does not significantly reduce in HFFc6, and even apparently increased in K562 when we used a smaller number of Hi-C reads. However, the small fractions of Micro-C loops (S12B Fig) for these down-sampled Hi-C show poor quality of those newly detected loops. Together, since our model is trained with high-resolution Hi-C as input, we recommend using at least 0.4B reads to generate Hi-C contact matrices.

To assess whether repeat random sampling in DDPM affects the robustness of our method, we repeated our prediction procedures four more times for HFFc6 and K562. The loops were called with Mustache (FDR = 0.05). We then made pairwise comparisons between the five predictions and found that the loop numbers and the loop sets are 100% equal. We also compared the top loops and still found that they are exactly equal. These results demonstrate that a random sample in DDPM does not affect HiC2MicroC, and we do not need to repeat sampling.

To investigate the role of the noise level in DDPM, we tested three more *β*_*T*_ values (0.1, 0.05, and 0.01). When we set *β*_*T*_ = 0.01, we can think of using an identical noise level during sampling (S2A Fig). We re-excuted our prediction procedures for K562 with each of the three values being used to determine the noise level of each iteration. The number of loops and fraction of recovering Micro-C loops are shown in S13 Fig. Large *β*_*T*_ results in more significant loops but does not affect fractions of Micro-C loops. The value (0.01) makes all noise levels of each iteration almost equal to one, resulting in the smallest loop number and fraction. In general, different continuous noise levels for each iteration in DDPM play a significant role in our model.

### 3.2 Anchors of loops bind CTCF in a convergent orientation

Loop extrusion is found to accompany CTCF binding in a convergent orientation [2,56]. In this section, we explore whether our loop anchors are assigned to a convergent orientation. For the four cell types (C42B, H1-ESC, K562, and HFFc6), we obtained the convergent orientation of CTCF binding sites at the anchors of chromatin loops using MotifFinder [41] in Juicer with only CTCF peaks as reference. The source for CTCF peaks can be found in S2 Table. There are four types of motif orientation: convergent (forward-reverse strand), divergent (reverse-forward), forward (forward-forward), and reverse (reverse-reverse) [2]. First, we report the fraction of all Mustache-detected loops that have convergent CTCF binding sites for each loop set in four cell types in S14A Fig, demonstrating that both HiC2MicroC and regression generate more CTCF-convergent loops than Micro-C and Hi-C in all testing cell types excluding H1-ESC.

We find the majority of CTCF-convergent loops are not shared between Micro-C and HiC2MicroC (S14B Fig) in K562. We perform APA on HiC2MicroC-specific CTCF-convergent loops (S14C Fig) on the four types of contact matrices (S14C Fig) and observe that these new CTCF-convergent loops are enriched for contact frequency, also in experimental Micro-C contact matrices, which indicates the ability of HiC2MicroC to discover more significant loops that Mustache misses from Micro-C. We can draw the same conclusion with regression-specific loops (S14D and S14E Fig).

**Fig 4:**
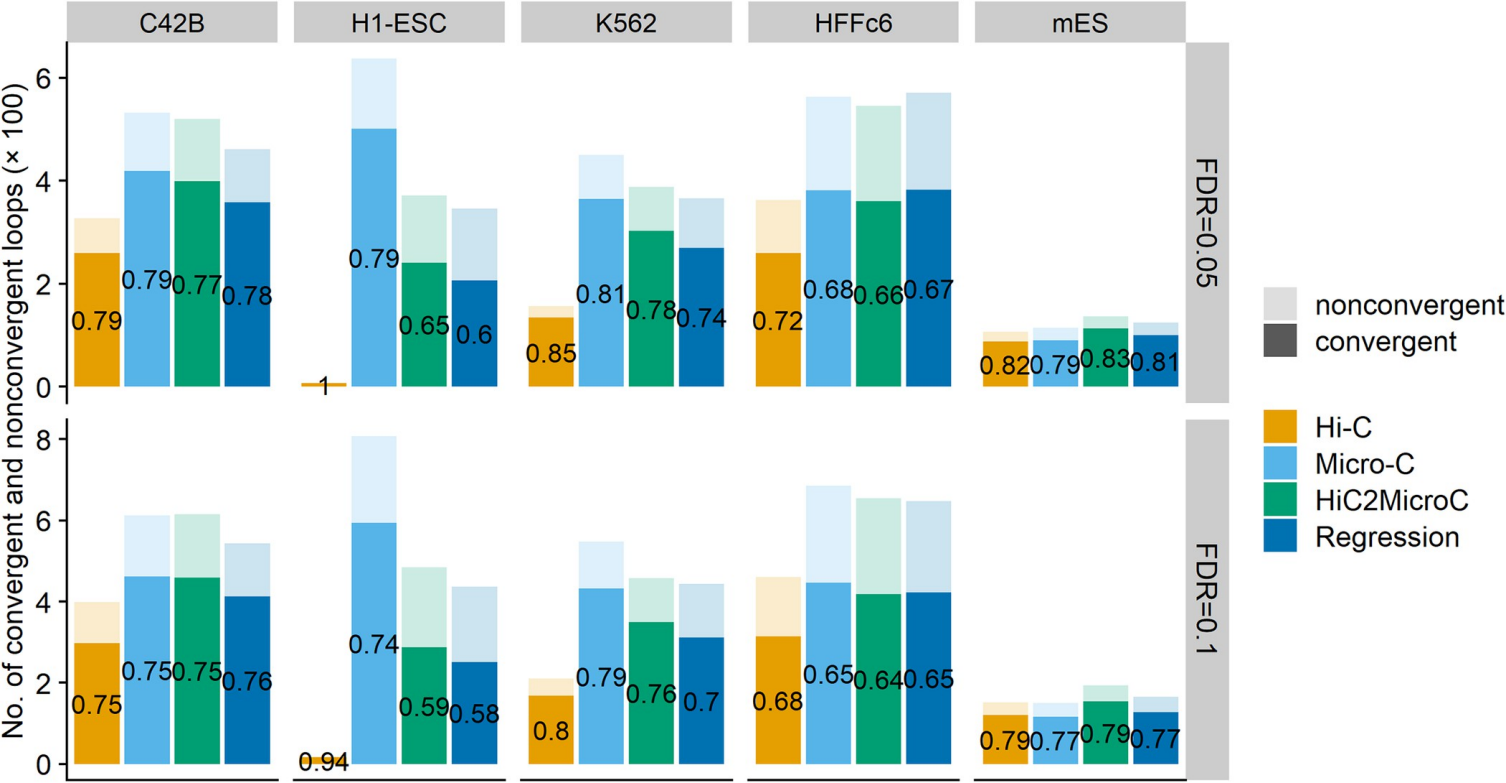
The fraction of top Mustache-detected loops that have convergent CTCF binding sites in four cell types.

Next, we examine the CTCF-convergent level of top loops. The evaluation results shown in Fig 4 indicate that HiC2MicroC outperforms regression by having more CTCF-convergent loops and higher percentages of top loops with convergent CTCF. Both HiC2MicroC and regression improve Hi-C by providing more CTCF-convergent loops, which are also consistent with Micro-C. Together, HiC2MicorC massively increases the number of loops with convergent CTCF.

### 3.3 Enrichment of structural proteins and histone modifications at loop loci

**Fig 5:**
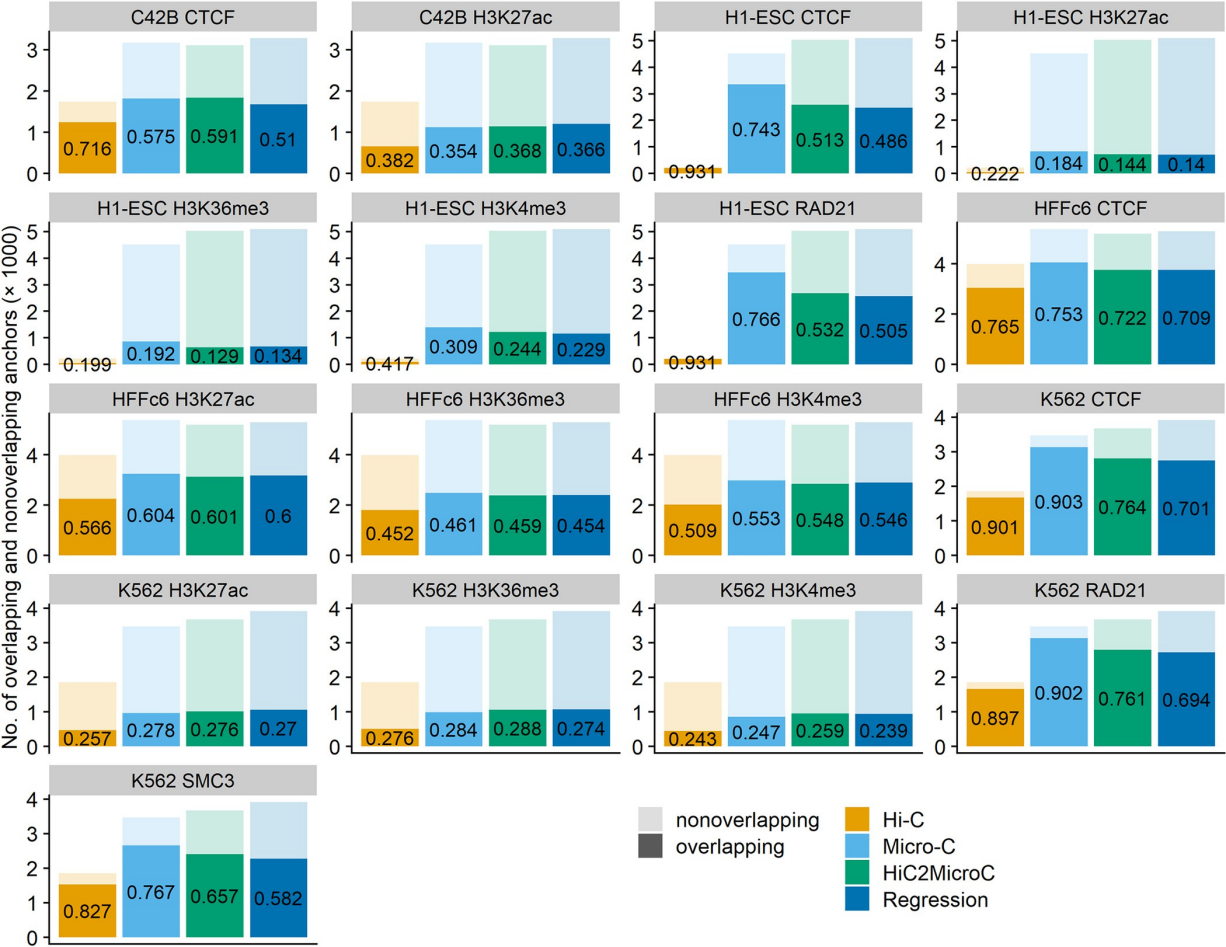
The fraction of anchors from top Mustache-detected loops (FDR = 0.1) overlapping structural proteins and histone modifications in four cell types.

CTCF and cohesin have been found to be enriched in loop loci [2]. In this paper, we define loop loci as all anchor sites of a loop set after removing duplicates, and the loop sets include Hi-C loops, Micro-C loops, top HiC2MicroC loops, and top regression loops. We test the presence of three structural proteins (CTCF, RAD21, and SMC3) and three important histone modifications (H3K27ac: a marker for active enhancers, H3K4me3 for the transcription start sites of active genes, and H3K36me3 associated with gene bodies) at the loop loci. The ChIP-seq data of these one-dimensional (1D) chromatin marks were downloaded from the website of ENCODE (details of the data source are in S2 Table). If a ChIP-seq peak overlaps an extended 15-kb anchor locus (5 kb anchor ± 5 kb extension), we then consider the anchor accompanies the corresponding structural proteins or histone modifications. HiC2MicroC outperforms regression by providing more overlapping anchors and higher percentages of anchors overlapping ChIP-seq peaks in almost all cell types (Fig 5 for FDR = 0.1 and S15 Fig for FDR = 0.05). Both HiC2MicroC and regression improve Hi-C by reporting more anchors that overlap with the peaks of ChIP-seq marks, and HiC2MicroC is the best method that has almost reached the same level as Micro-C.

### 3.4 Evaluating loops that link enhancers and promoters

**Fig 6:**
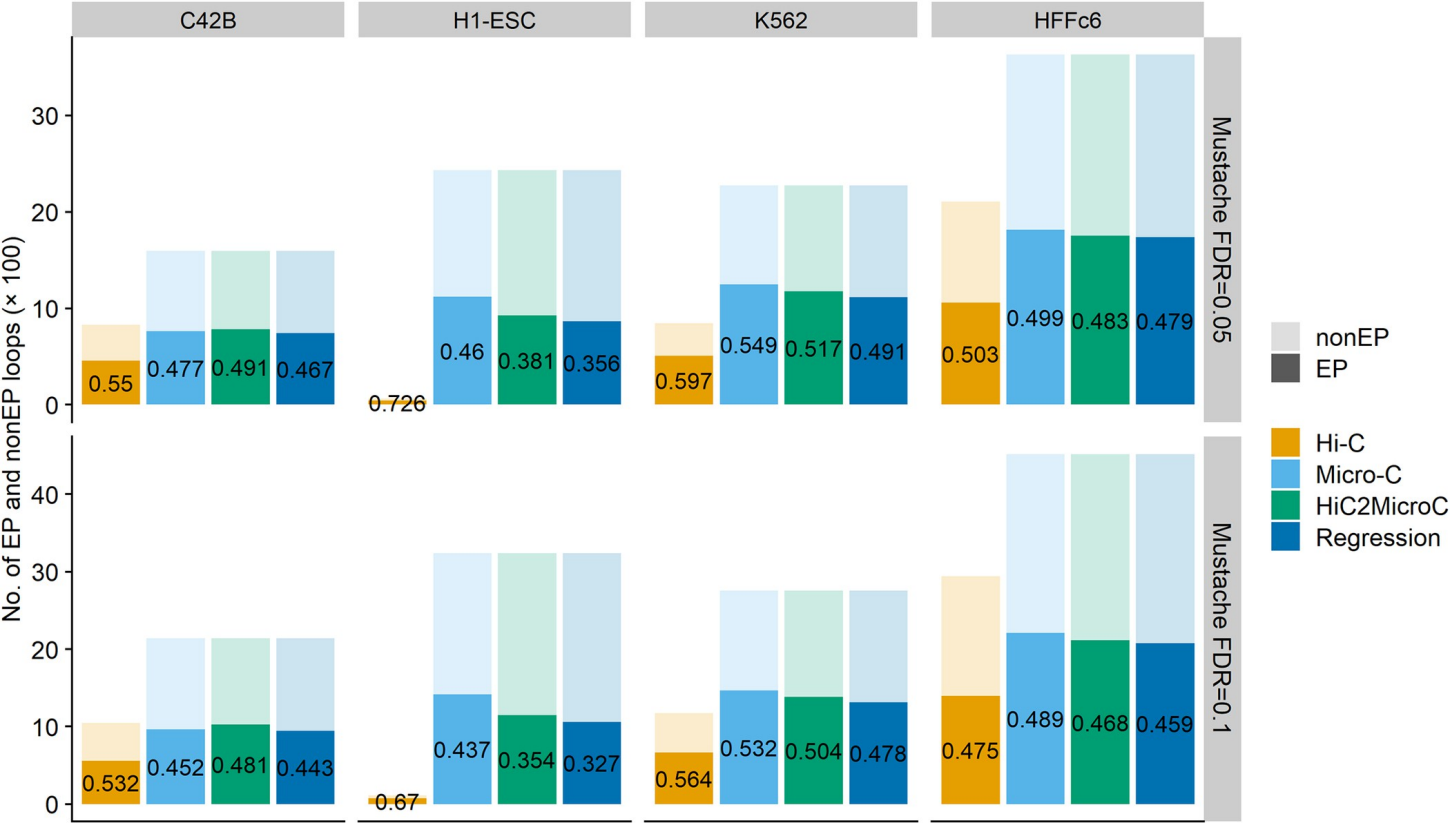
The percentage of top Mustache-detected loops that can be labelled as EP loops in four cell lines. The universal chromatin state annotation is used to demarcate promoter and enhancer regions.

In this section, we examine whether the loops are associated with two functional regulatory elements (promoters and enhancers). We consider a loop as an enhancer-promoter (EP) loop if one anchor of the loop overlaps with at least one enhancer and the other anchor overlaps with at least one promoter (anchors still extended to 15 kb). Each loop set can be classified into two categories (EP and non-EP). We use a universal chromatin state annotation [57] to obtain promoter and enhancer regions. The higher percentages of top Mustache-detected HiC2MicroC loops (Fig 6) that are labeled as EP loops demonstrate that HiC2MicroC can recover more EP loops than the regression method in all four cell types. In C42B, HiC2MicroC achieves even higher percentages than Micro-C.

Furthermore, we downloaded cell-type-specific chromatin states (build hg19) for H1-ESC and K562 from http://hgdownload.soe.ucsc.edu/goldenPath/hg19/encodeDCC/wgEncodeBroadHmm/, which were further converted to the reference build hg38 using liftOver. The evaluation results (S16 Fig) also show that HiC2MicroC outperforms regression in two cell types (H1-ESC and K562) by recovering more EP loops. Taken together, HiC2MicroC annotates a comparable or higher number of EP loops with Micro-C, which may correlate with gene regulation.

Finally, we evaluated our predicted Micro-C against the EP loops detected by Perturb-seq (S17 Fig). The 81 Perturb-seq EP loops were obtained from S3 Table in [58]. The enhancer annotation was converted to hg38 with liftOver. We extracted interaction frequencies of pixels overlapping 81 EP loops from Hi-C, Micro-C, and predicted Micro-C (HiC2MicroC and regression). The boxplots shown in S17 Fig indicate that both HiC2MicroC and regression elevate interaction frequencies from Hi-C for these 81 EP loops detected by Perturb-seq, and the changes are statistically significant.

### 3.5 Evaluating loops on PCMicro-C and CTCF ChIA-PET

**Fig 7:**
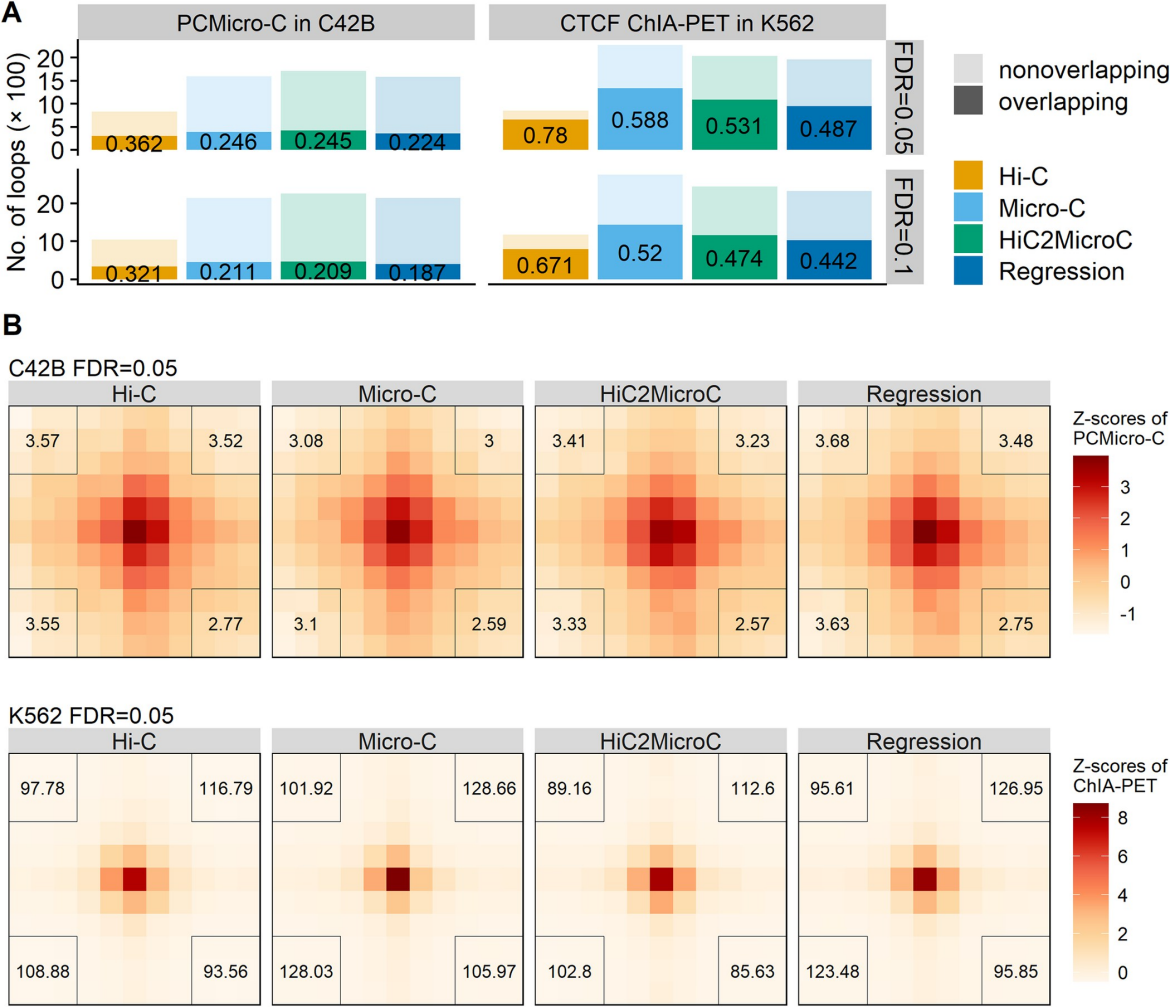
(A) Recovering PCMicro-C and CTCF ChIA-PET loops by Mustache-detected loops from Hi-C, Micro-C, top HiC2MicroC, and top regression. (B) APA plots of Hi-C and Micro-C loops (FDR = 0.05) on PCMicro-C contact matrices in C42B. (C) APA plots of Hi-C and Micro-C loops (FDR = 0.05) on CTCF ChIA-PET contact matrices in K562.

We further benchmark the methods on the loops that are called on Hi-C and Micro-C with non-Hi-C data sets as reference. We first explore whether loops found in Hi-C and Micro-C are shared with loops detected in PCMicro-C and ChIA-PET mediated by CTCF (sources for loops and contact matrices in S3 Table). We compute the percentages of Hi-C and Micro-C loops overlapping PCMicro-C and CTCF ChIA-PET loops. The extending size for checking whether two loops match is set to one. HiC2MicroC recovers more PCMicro-C and CTCF ChIA-PET loops than regression, and both exceed Hi-C, even though Hi-C has the highest percentage (Fig 7A). Specifically, 58.8% Micro-C loops (FDR = 0.05) are also found in CTCF ChIA-PET loops in K562, followed by 53.1% top HiC2MicroC loops and 48.7% top regression loops.

We next performed APA of Hi-C and Micro-C plots on PCMicro-C and CTCF ChIA-PET contact matrices. The APA heat maps are shown in Fig 7B and 7C for FDR = 0.05 and S18 Fig for FDR = 0.1. The APA scores for HiC2MicroC and regression are consistently larger than the corresponding scores for Hi-C as evidence suggesting our methods improve Hi-C. Some of the APA scores of HiC2MicroC are even larger than the corresponding scores of Micro-C.

Since the human cell type GM12878 has been extensively studied in 3D genome structures, we also benchmark the methods on GM12878. Specifically, we execute our methods (HiC2MicroC and regression) on its Hi-C data to predict Micro-C and then detect loops on Hi-C and predicted Micro-C with Mustache. The number of loops for Hi-C, HiC2MicroC, and regression detected by Mustache with FDR = 0.05 is 1,148, 5,682, and 6,918, respectively. The corresponding number of loops with FDR = 0.1 is 1,548, 7,640, and 9,178. Since we do not know the number of Micro-C loops of GM12878 (no Micro-C data published on GM12878), we use all loops of HiC2MicroC and regression in the following analysis. We first examine the ability to recover CTCF ChIA-PET loops of GM12878. Even though regression owns more Mustache-detected loops, its percentage of loops is smaller than HiC2MicroC (S19A Fig), indicating that HiC2MicroC has a greater ability to recover CTCF ChIA-PET loops. We also generate APA plots of Hi-C and predicted-Micro-C loops on CTCF ChIA-PET contact matrices in GM12878 (S19B Fig), showing that the APA scores of HiC2MicroC are higher than those of regression.

### 3.6 Specific examples for recovering Micro-C loops

**Fig 8:**
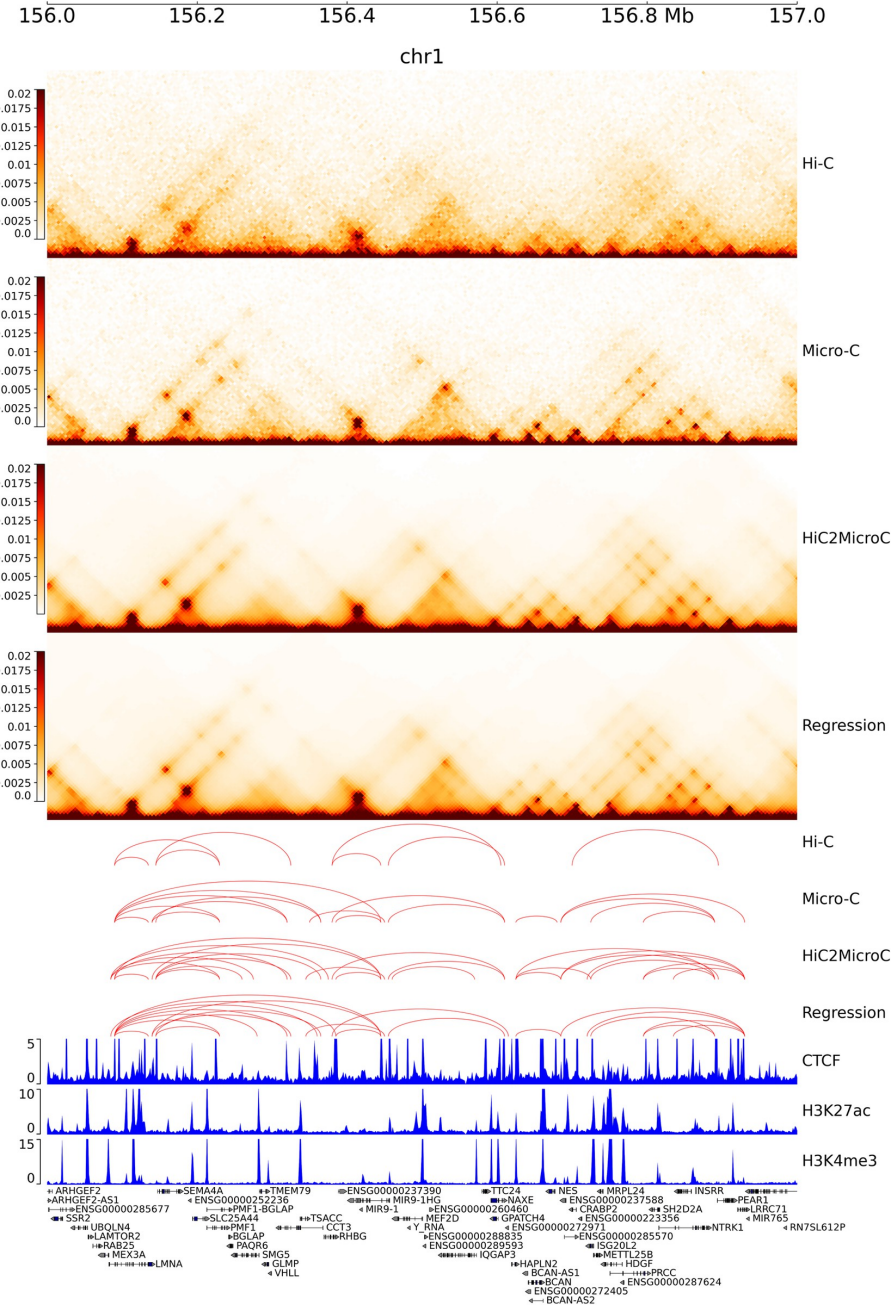
From top to down: the contact matrices of Hi-C, Micro-C, and HiC2MicroC- and regression-predicted Micro-C, the Mustache-detected loops (FDR = 0.05) of Hi-C, Micro-C, HiC2MicroC, and regression, the ChIP-seq signals for CTCF, H3K27ac, and H3K4me3, and gene annotations in K562.

In this section, we use two selected chromatin regions in K562 and H1-ESC to demonstrate the effectiveness of HiC2MicroC in recovering Micro-C loops. The genome tracks (Figs 8 and S20–S22) were generated using pyGenomeTracks (v3.6) [59], including contact matrices, loop links shown by red arcs, ChIP-seq signals, and gene annotations. In the first region 156–157 Mb on chromosome 1 in K562 (Fig 8), Hi-C only annotates eight loops, whereas Micro-C results in 17 loops distributed across the region. Both HiC2MicroC and regression accurately recover most of the Hi-C and Micro-C loops and identify some additional loops, which are supported by CTCF, H3K27ac, and H3K4me3 peaks. Another example on the same chromosome in K562 (S20 Fig) shows that HiC2MicroC recovered more Micro-C loops than regression.

Furthermore, we provide two regions (6–7 Mb and 9–10 Mb) on chromosome 1 in H1-ESC (S21–S22 Fig). We did not find any Hi-C loop in the first region and observed two Hi-C loops in the second region. Despite that the Hi-C matrix is too sparse to provide useful information, after being input into HiC2MicroC almost all of the Micro-C loops are found (S21–S22 Fig). Regression also recovers part of Micro-C loops, but not as good as HiC2MicroC. Overall, these two specific examples suggest that HiC2MicroC can successfully recover Micro-C loops even though we can hardly detect any loop from the corresponding Hi-C.

### 3.7 Recovering Micro-C loops at the 1-kb resolution

**Fig 9:**
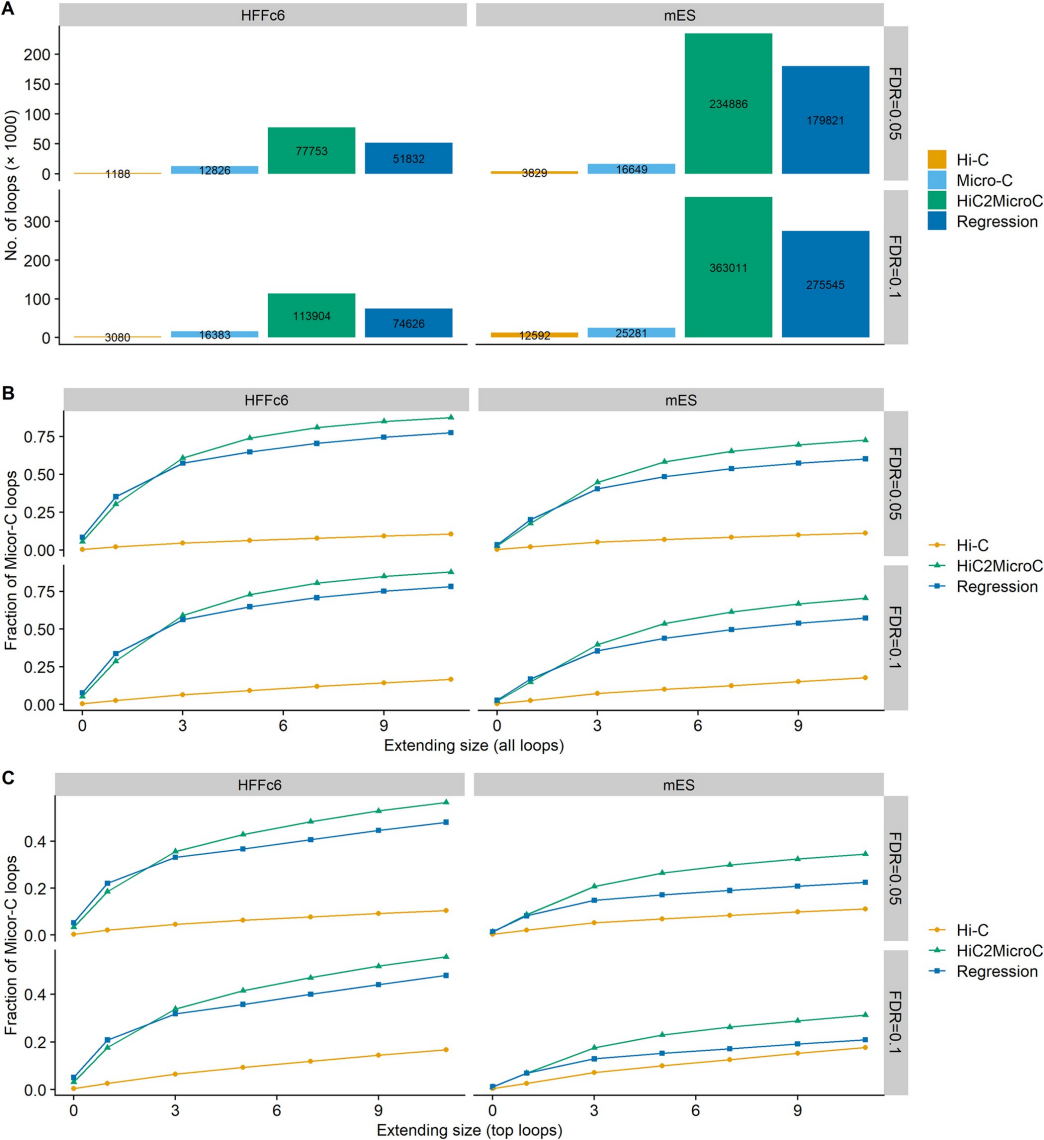
(A) The number of loops identified by Mustache on Hi-C, Micro-C, HiC2MicroC, and regression matrices at 1-kb resolution in two cell types. (B) The fraction of recovering Micro-C loops for all Mustache-detected loops from HiC2MicroC and regression with Hi-C loops as baselines. (C) The same as (B) but with top loops.

In the previous sections, we evaluate loops called on 5-kb contact matrices. In this section, we assess the methods with loops called on 1-kb contact matrices. It is worth mentioning that identifying loops on 1-kb Hi-C contact matrices is still a challenging task to date [3,21]. Since the average fragment length is 4 kb in Hi-C assay, we need deeply sequenced Hi-C reads to reach 1 kb resolution. Another major concern is runtime. Compared with training for 5-kb maps that takes less than a day, training for 1-kb takes more than seven days. Prediction for 1 kb maps also takes a much longer time because of the extreme increase of the samples. Considering the sequencing depths of the cell types, we only ran our methods for two types (HFFc6 and mESCs). The number of loops detected by Mustache is shown in Fig 9A. Contrary to what we observe in Fig 2A, HiC2MicroC results in more loops than regression in both cell types. We next computed the percentage of recovering Micro-C loops. As previously found in Fig 2, HiC2MicroC achieves a higher fraction either on all loops (Fig 9B) or on top loops (Fig 9C) when the extending size is larger than one, indicating that compared with regression, HiC2MicroC is better at recovering Micro-C loops. The APA plots (S23 and S24 Figs) also indicate that both HiC2MicroC and regression have made Hi-C closer to experimental Micro-C.

## Discussion

Hi-C contact maps have been widely used to exhibit genome-wide chromatin looping phenomenon. However, due to the longer average fragment generated by restriction enzymes, Hi-C has a relatively lower signal-to-noise ratio than its variant Micro-C, which replaces restriction enzymes with MNase. However, compared with Hi-C, the number of available Micro-C datasets in the literature is much smaller. To make full use of Hi-C datasets, we present HiC2MicroC, a Hi-C-conditioned generative DDPM for predicting Micro-C based on Hi-C. To demonstrate the effectiveness of HiC2MicroC, we also train regression models to directly learn the mapping relationships between Hi-C and Micro-C. We use Hi-C and Micro-C of six cell types to evaluate our methods. We first show that both HiC2MicroC and regression can successfully recover genome-wide and specific Micro-C loops, and HiC2MicroC has larger recovering fractions than regression in almost all cell types. Next, we report that the majority of our recovered loops are anchored at CTCF binding sites in a convergent orientation. HiC2MicroC outperforms regression by annotating more loops with convergent CTCF. Furthermore, we demonstrate that anchors of HiC2MicroC-recovered loops are more enriched for structural proteins and epigenetic features than anchors from regression. Compared with regression and Hi-C, the number of HiC2MicroC loops that link promoters and enhancers is more comparable to those from Micro-C. We also show that loops from HiC2MicroC are more consistent with loops from PCMicro-C and CTCF ChIA-PET than regression. In conclusion, HiC2MicroC is an effective method for learning and then predicting Micro-C only based on Hi-C and can be a valuable tool for further exploring Hi-C data.

## Supporting information

S1 Table The source of Hi-C and Micro-C datasets. (DOCX)

S2 Table The source of ChIP-seq datasets. (XLSX)

S3 Table The source of PCMicro-C and ChIA-PET datasets. (DOCX)

S1 Fig (A) The U-Net architecture used in HiC2MicroC (the numbers denote the shape of the output tensor *C*×*H*×*W*). (B) The residual block with the embeddings of the noise level added after the first normalization layer. (C) The attention block. (D) The down-sampling and up-sampling blocks in U-Net.
(TIF)

S2 Fig (A) The noise scale curves for different combinations of *T*−*β*_1_−*β*_*T*_. (B) The corresponding validation L1 loss of different combinations. (C) The loss curves for training and validation of HiC2MicroC and regression at 5-kb resolution.
(TIFF)

S3 Fig (A) The number of loops identified by SIP on Hi-C, Micro-C, HiC2MicroC, and regression matrices. (B) The fraction of recovering SIP loops for all SIP-detected loops for Hi-C, HiC2MicroC, and regression.
(TIFF)

S4 Fig The loop length distribution. For HiC2MicroC and regression, we use top Mustache-detected loops.
(TIFF)

S5 Fig APA plots for all Mustache-detected Micro-C loops (FDR = 0.05) on matrices of Hi-C, Micro-C, HiC2MicroC, and regression in five cell types. The APA scores are shown at each corner. The number of loops used for generating APA plots is also provided beside cell type names.
(TIFF)

S6 Fig APA plots for all SIP-detected Micro-C loops (FDR = 0.05) on matrices of Hi-C, Micro-C, HiC2MicroC, and regression in five cell types. The APA scores are shown at each corner. The number of loops used for generating APA plots is also provided beside cell type names.
(TIFF)

S7 Fig APA plots for all SIP-detected Micro-C loops (FDR = 0.01) on matrices of Hi-C, Micro-C, HiC2MicroC, and regression in five cell types. The APA scores are shown at each corner. The number of loops used for generating APA plots is also provided beside cell type names.
(TIFF)

S8 Fig APA plots for Mustache-detected Micro-C-specific loops (FDR = 0.1) on matrices of Hi-C, Micro-C, HiC2MicroC, and regression in five cell types. The APA scores are shown at each corner. The number of loops used for generating APA plots is also provided beside cell type names.
(TIFF)

S9 Fig APA plots for Mustache-detected Micro-C-specific loops (FDR = 0.05) on matrices of Hi-C, Micro-C, HiC2MicroC, and regression in five cell types. The APA scores are shown at each corner. The number of loops used for generating APA plots is also provided beside cell type names.
(TIFF)

S10 Fig APA plots for SIP-detected Micro-C-specific loops (FDR = 0.05) on matrices of Hi-C, Micro-C, HiC2MicroC, and regression in five cell types. The APA scores are shown at each corner. The number of loops used for generating APA plots is also provided beside cell type names.
(TIFF)

S11 Fig APA plots for SIP-detected Micro-C-specific loops (FDR = 0.01) on matrices of Hi-C, Micro-C, HiC2MicroC, and regression in five cell types. The APA scores are shown at each corner. The number of loops used for generating APA plots is also provided beside cell type names.
(TIFF)

S12 Fig (A) The number of loops detected by Mustache (FDR = 0.05) with four types of Hi-C as input (original and three down-sampling datasets) in HFFc6 and K562. (B) The fraction of recovering Micro-C loops for top Mustache-detected loops from HiC2MicroC-predicted Micro-C with four types of Hi-C as input.
(TIFF)

S13 Fig (A) The number of loops detected by Mustache (FDR = 0.05) in K562 from HiC2McroC-predicted Micro-C with four *β*_*T*_ values (0.95, 0.1, 0.05, and 0.01) to determine noise level. (B) the Fraction of recovering Micro-C loops.
(TIFF)

S14 Fig (A) The fraction of all Mustache-detected loops that have convergent CTCT binding sites. (B) The agreement of convergent loops between Micro-C and HiC2MicroC. (C) APA plots of HiC2MicroC-specific, CTCF-convergent loops (FDR = 0.05) on the four types of contact matrices. (D) The agreement of convergent loops between Micro-C and regression. (E) APA plots of regression-specific, CTCF-convergent loops (FDR = 0.05) on the four types of contact matrices.
(TIFF)

S15 Fig The fraction of anchors from top Mustache-detected loops (FDR = 0.05) overlapping structural proteins and histone modifications in four cell types. (TIFF)

S16 Fig The percentage of top Mustache-detected loops that can be labelled as EP loops in two cell types. The cell-specific chromatin state annotation is used to demarcate promoter and enhancer regions.
(TIFF)

S17 Fig The corresponding contact frequencies of 81 Perturb-seq EP loops in K562. P-values are computed with the student’s *t-*test.
(TIFF)

S18 Fig Top: APA plots of Hi-C and Micro-C loops (FDR = 0.1) on PCMicro-C contact matrices in C42B. Bottom: APA plots of Hi-C and Micro-C loops (FDR = 0.1) on CTCF ChIA-PET contact matrices in K562.
(TIFF)

S19 Fig (A) Recovering CTCF ChIA-PET loops by Mustache-detected loops from Hi-C, HiC2MicroC, and regression. (B) APA plots of Hi-C and predicted-Micro-C loops within genomic distance [100kb-2Mb] on CTCF ChIA-PET contact matrices in GM12878.
(TIFF)

S20 Fig From top to down: the contact matrices of Hi-C, Micro-C, and HiC2MicroC- and regression-predicted Micro-C, the Mustache-detected loops (FDR = 0.05) of Hi-C, Micro-C, HiC2MicroC, and regression, the ChIP-seq signals for CTCF, H3K27ac, and H3K4me3, gene annotations in K562. (TIFF)

S21 Fig From top to down: the contact matrices of Hi-C, Micro-C, and HiC2MicroC- and regression-predicted Micro-C, the Mustache-detected loops (FDR = 0.05) of Hi-C, Micro-C, HiC2MicroC, and regression, the ChIP-seq signals for CTCF, H3K27ac, and H3K4me3, gene annotations on chromosome 1 (6–7 Mb) in H1-ESC. (TIFF)

S22 Fig From top to down: the contact matrices of Hi-C, Micro-C, and HiC2MicroC- and regression-predicted Micro-C, the Mustache-detected loops (FDR = 0.05) of Hi-C, Micro-C, HiC2MicroC, and regression, the ChIP-seq signals for CTCF, H3K27ac, and H3K4me3, gene annotations on chromosome 1 (9–10 Mb) in H1-ESC. (TIFF)

S23 Fig APA plots of all Mustache-detected Micro-C loops from Hi-C and Micro-C, HiC2MicroC, and regression. All loops are detected on 1-kb contact matrices.
(TIFF)

S24 Fig APA plots of Mustache-detected Micro-C-specific loops from Hi-C and Micro-C, HiC2MicroC, and regression. Specific loops are generated with the extending size set to four by filtering out loops that are found in Hi-C loops. All loops are detected on 1-kb contact matrices.
(TIFF)
