# Supplementary material for: Learning Micro-C from Hi-C with diffusion models
Source: PLoS Comput Biol. 2024 May 17;20(5):e1012136. doi: 10.1371/journal.pcbi.1012136 (PMC11139321; doi:10.1371/journal.pcbi.1012136)
Supplement: S1 Table — (DOCX) [file pcbi.1012136.s001.docx]

**Table S1.** Data source for Hi-C and Micro-C.

| Cell type | Genome build | Assay | Resolutions | Source | No. of valid pairs |
| --- | --- | --- | --- | --- | --- |
| HFFc6 | hg38 | Hi-C | 5 kb, 1 kb | 4D Nucleome, 4DNES2R6PUEK | 2.9B |
|  |  | Micro-C | 5 kb, 1 kb | 4D Nucleome, 4DNESWST3UBH | 5.9B |
| H1-ESC | hg38 | Hi-C | 5 kb | 4D Nucleome, 4DNESRJ8KV4Q | 0.4B |
|  |  | Micro-C | 5 kb | 4D Nucleome, 4DNES21D8SP8 | 3.2B |
| K562 | hg38 | Hi-C | 5 kb | GEO, GSE63525 | 0.8B |
|  |  | Micro-C | 5 kb | GEO, GSE206131 | 1.4B |
| C42B | hg38 | Hi-C | 5 kb | GEO, GSE204996 | 1.1B |
|  |  | Micro-C | 5 kb | GEO, GSE204997 | 3.0B |
| mESC | mm10 | Hi-C | 5kb, 1kb | GEO, GSE96107 | 2.6B |
|  |  | Micro-C | 5kb, 1kb | GEO, GSE130275 | 2.6B |
| GM12878 | hg38 | Hi-C | 5kb | GEO, GSE63525 | 2.9B |
