# Supplementary material for: Learning Micro-C from Hi-C with diffusion models
Source: PLoS Comput Biol. 2024 May 17;20(5):e1012136. doi: 10.1371/journal.pcbi.1012136 (PMC11139321; doi:10.1371/journal.pcbi.1012136)
Supplement: S3 Table — (DOCX) [file pcbi.1012136.s003.docx]

**Table S3.** Data source for PCMicro-C and ChIA-PET

| Cell type | Genome build | Assay | Data type | Source |
| --- | --- | --- | --- | --- |
| C42B | hg38 | PCMicro-C | loops | Table S3J [1] |
|  |  |  | hic | GEO, GSE204995 |
| K562 | hg38 | CTCF ChIA-PET | loops | ENCODE, ENCFF607PZX |
|  |  |  | hic | ENCODE, ENCFF436ONK |
| GM12878 | hg38 | CTCF ChIA-PET | loops | ENCODE, ENCFF780PGS |
|  |  |  | hic | ENCODE, ENCFF379AWZ |

**References**

1. Lee BH, Wu Z, Rhie SK. Characterizing chromatin interactions of regulatory elements and nucleosome positions, using Hi-C, Micro-C, and promoter capture Micro-C. Epigenetics Chromatin. 2022;15(1):41. <https://doi.org/10.1186/s13072-022-00473-4> PMID: 36544209
