# Supplementary figures and images for: Learning Micro-C from Hi-C with diffusion models
Source: PLoS Comput Biol. 2024 May 17;20(5):e1012136. doi: 10.1371/journal.pcbi.1012136 (PMC11139321; doi:10.1371/journal.pcbi.1012136)

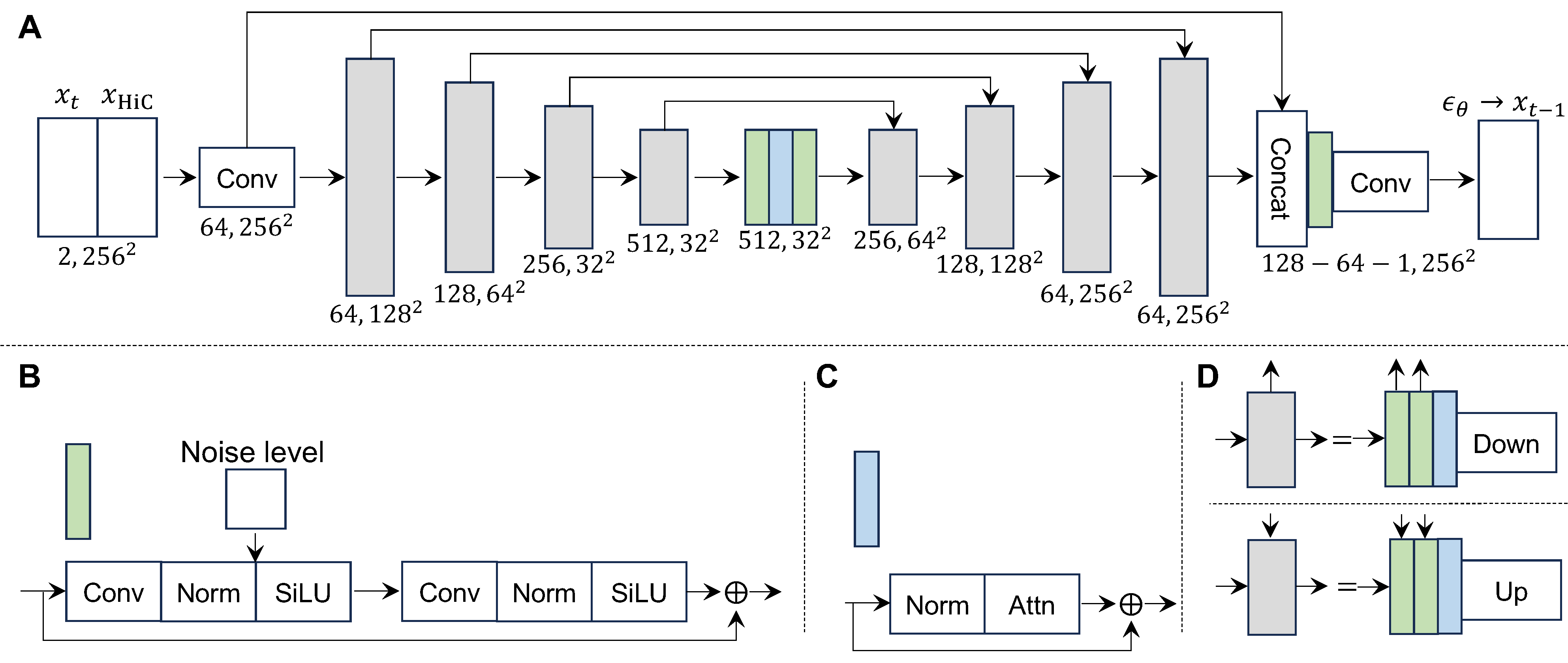

Supplement: S1 Fig — (A) The U-Net architecture used in HiC2MicroC (the numbers denote the shape of the output tensor C×H×W). (B) The residual block with the embeddings of the noise level added after the first normalization layer. (C) The attention block. (D) The down-sampling and up-sampling blocks in U-Net. (TIF) [file pcbi.1012136.s004.tif]

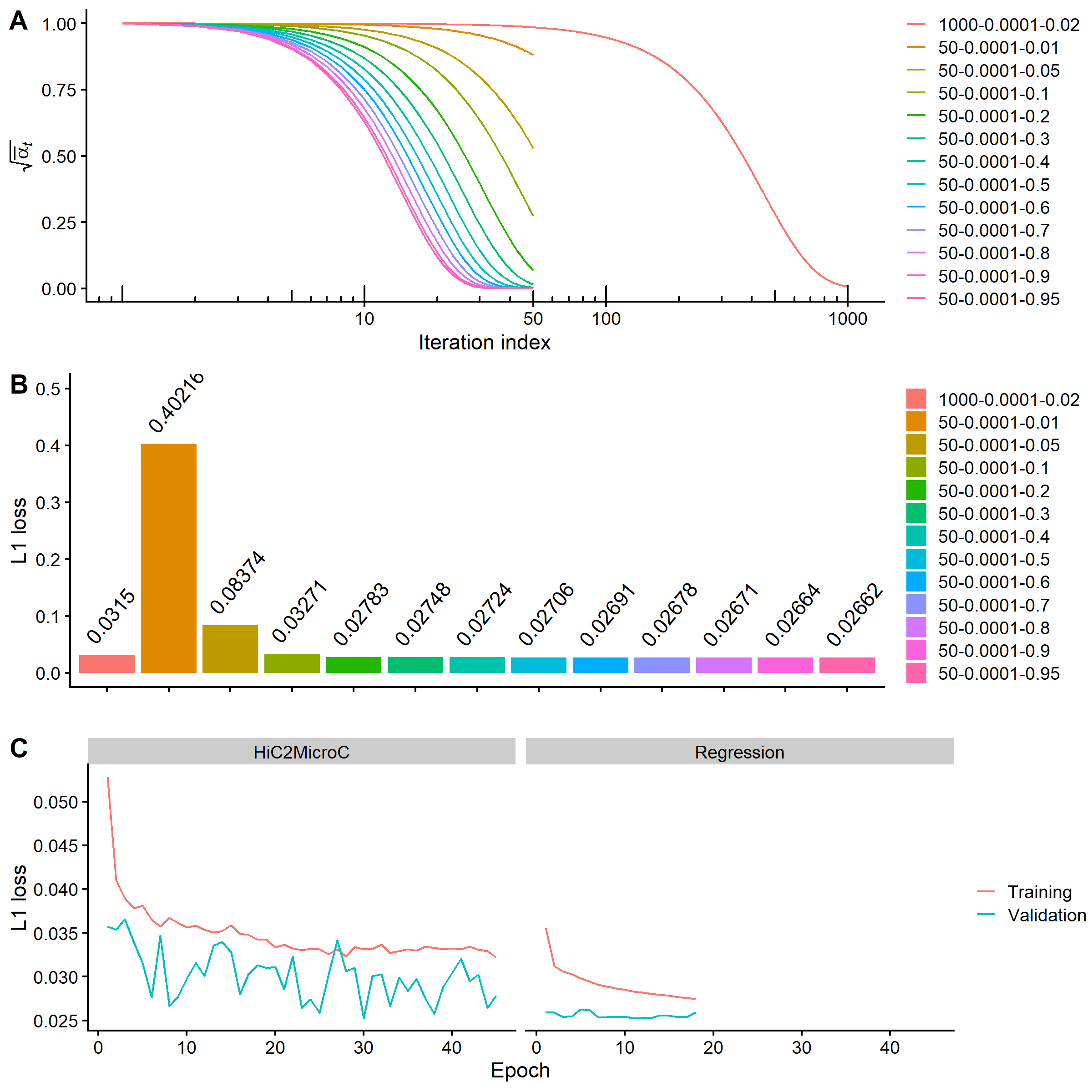

Supplement: S2 Fig — (A) The noise scale curves for different combinations of T−β1−βT. (B) The corresponding validation L1 loss of different combinations. (C) The loss curves for training and validation of HiC2MicroC and regression at 5-kb resolution. (TIFF) [file pcbi.1012136.s005.tiff]

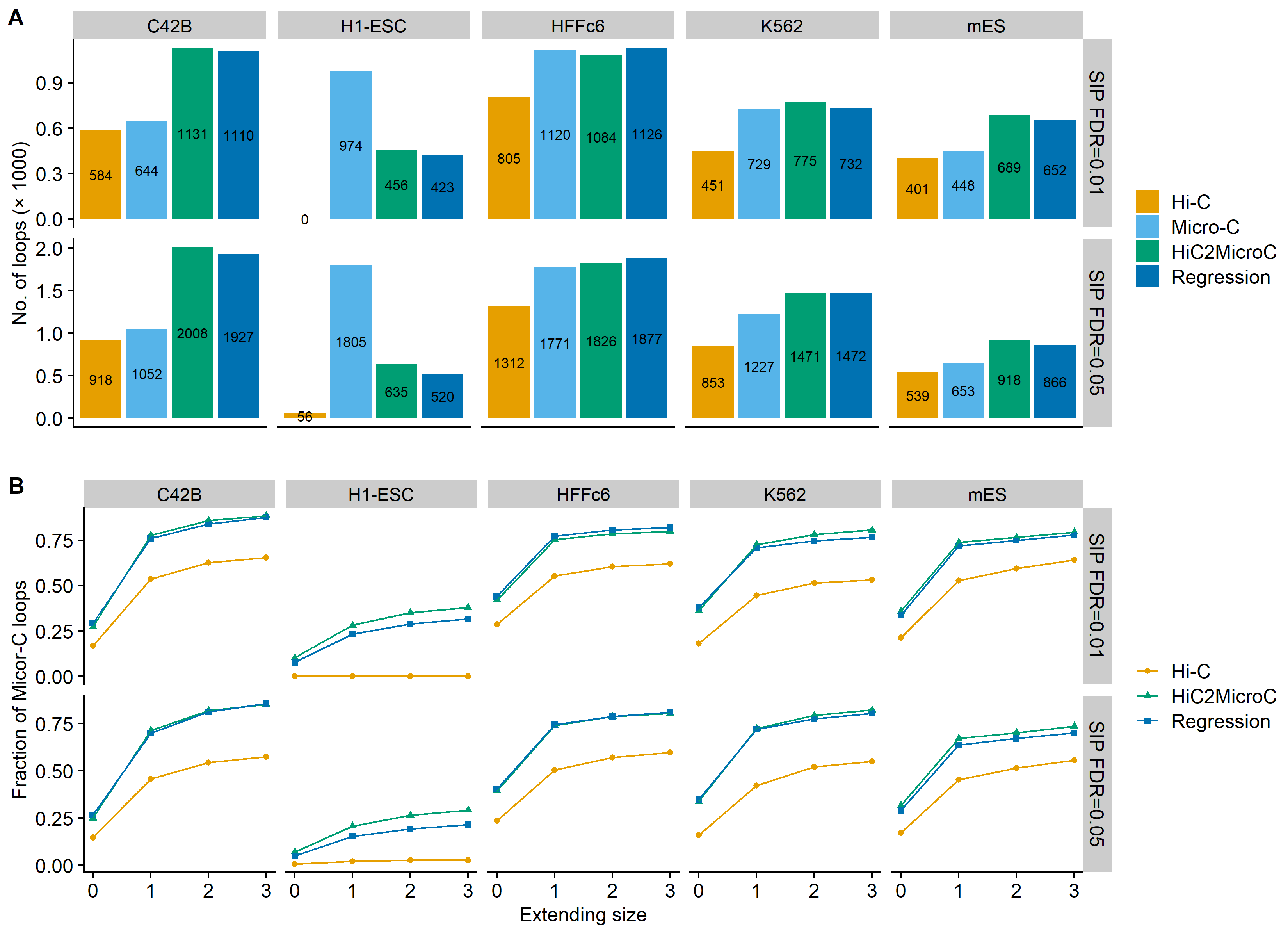

Supplement: S3 Fig — (A) The number of loops identified by SIP on Hi-C, Micro-C, HiC2MicroC, and regression matrices. (B) The fraction of recovering SIP loops for all SIP-detected loops for Hi-C, HiC2MicroC, and regression. (TIFF) [file pcbi.1012136.s006.tiff]

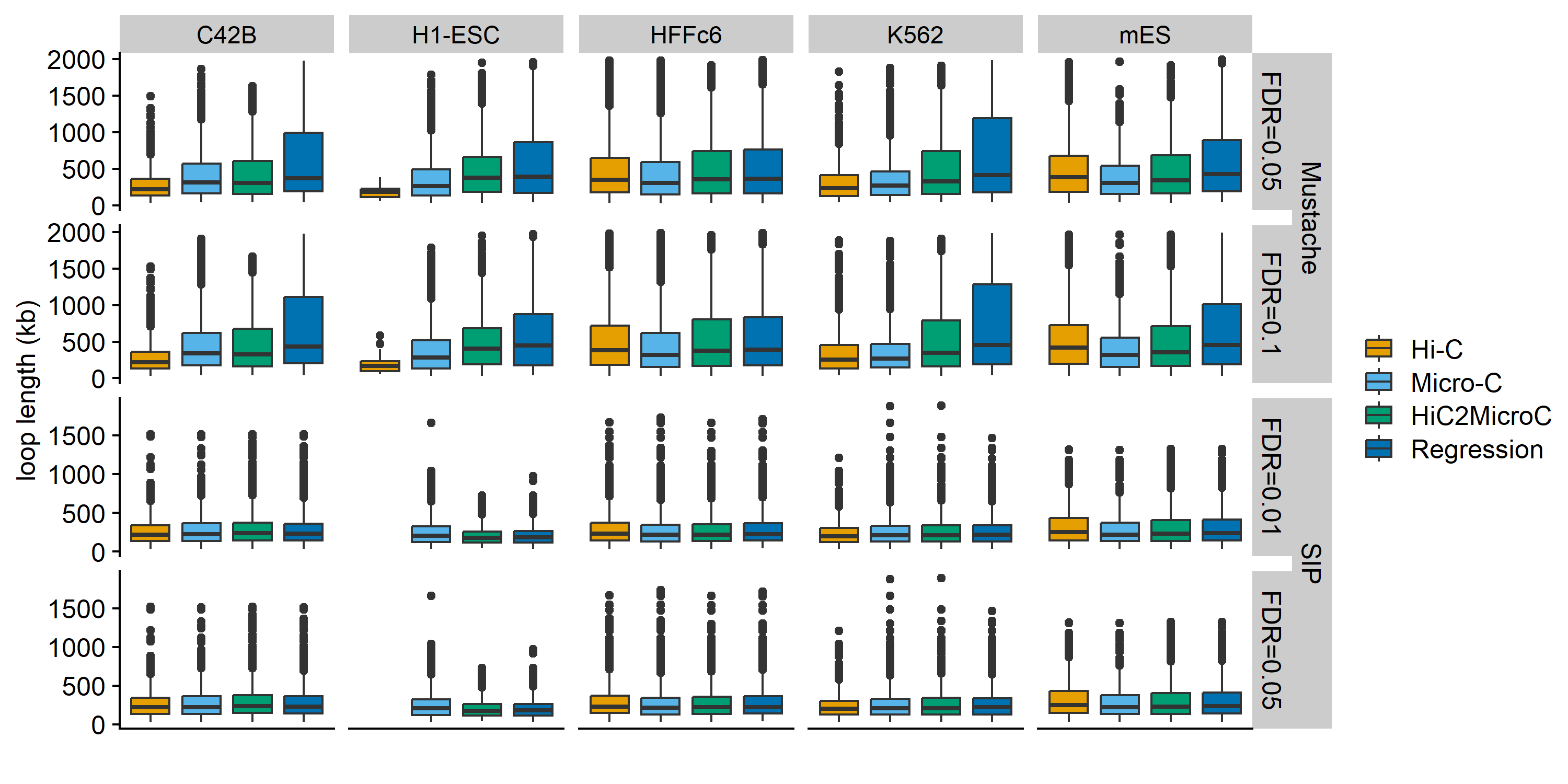

Supplement: S4 Fig — For HiC2MicroC and regression, we use top Mustache-detected loops. (TIFF) [file pcbi.1012136.s007.tiff]

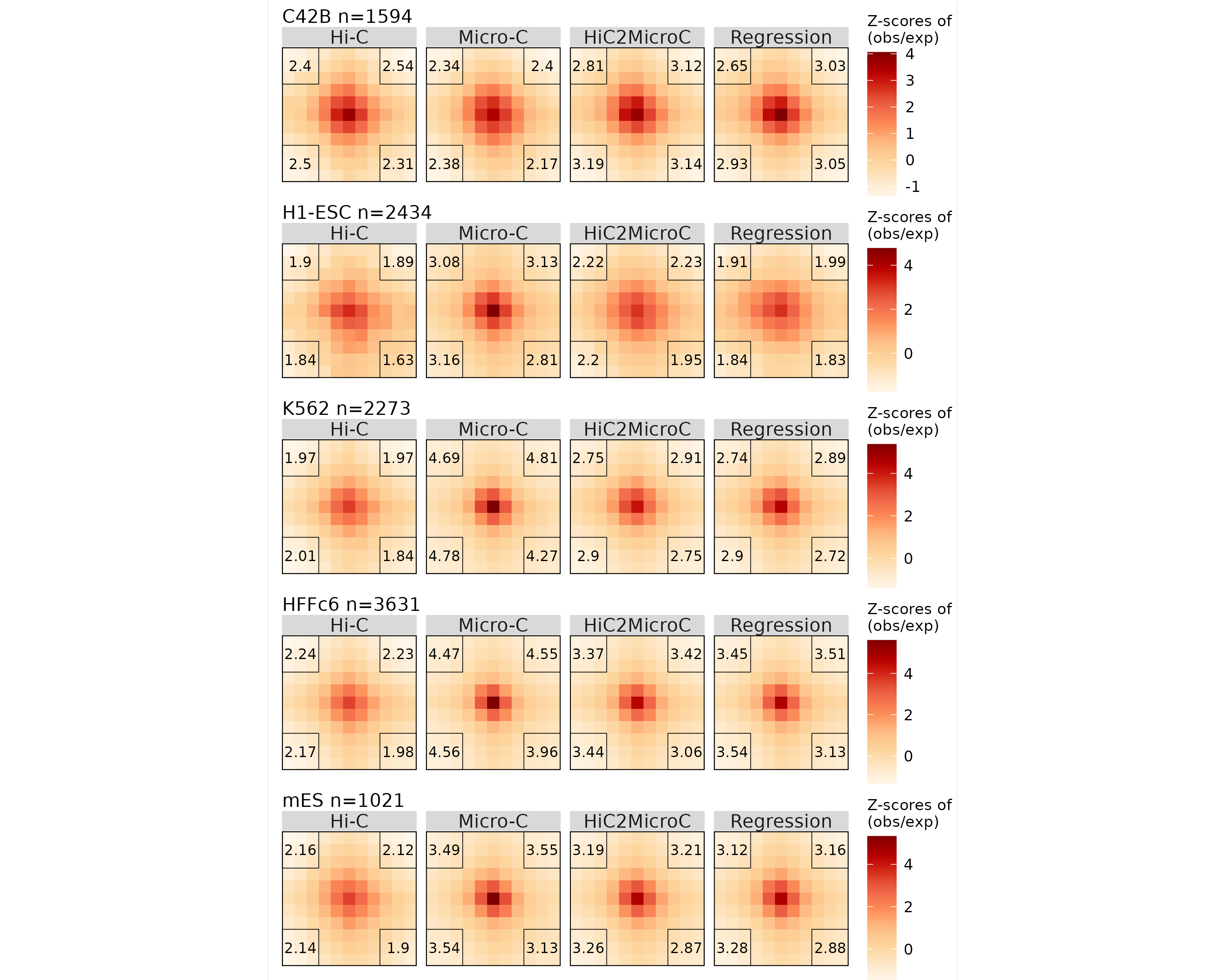

Supplement: S5 Fig — The APA scores are shown at each corner. The number of loops used for generating APA plots is also provided beside cell type names. (TIFF) [file pcbi.1012136.s008.tiff]

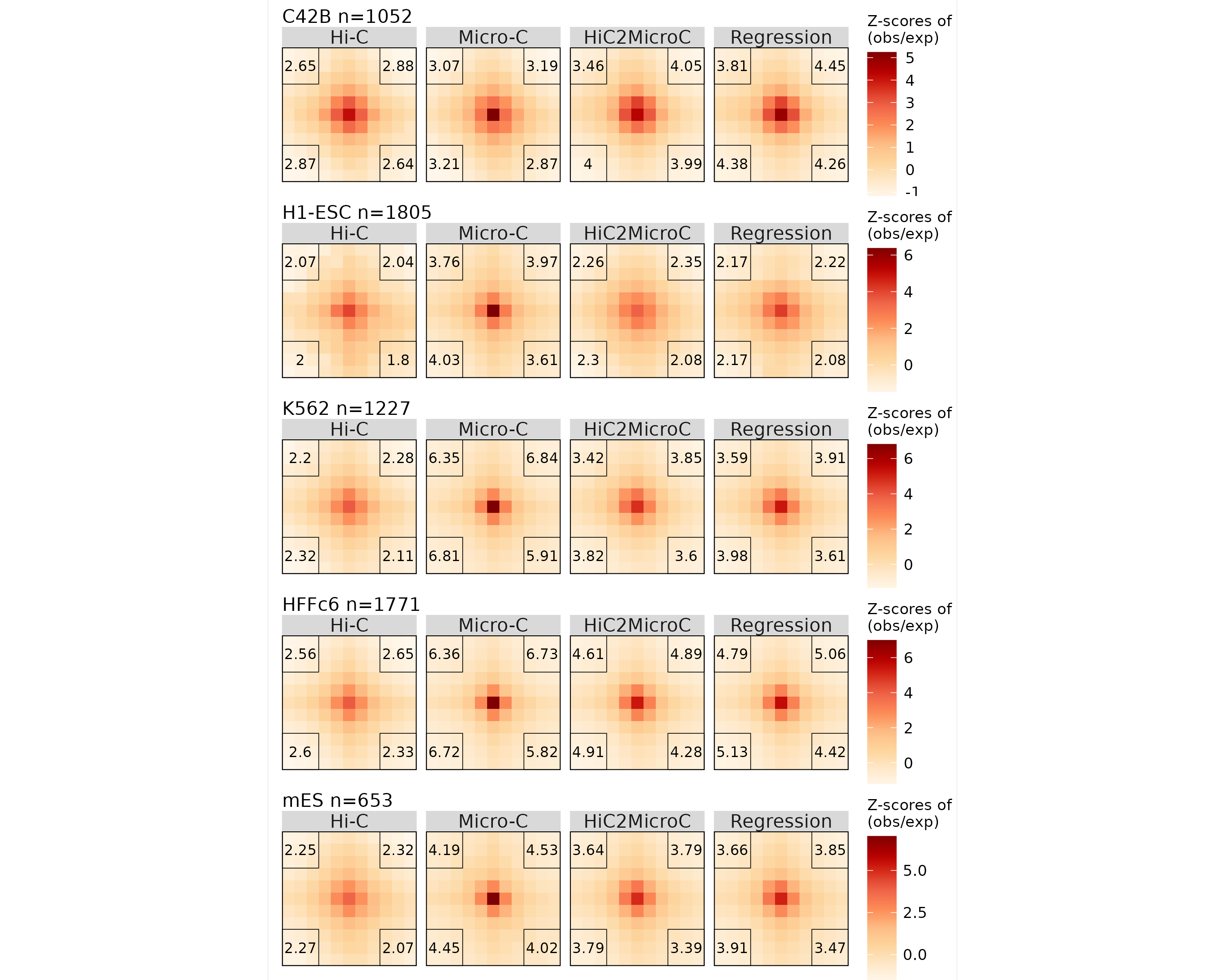

Supplement: S6 Fig — The APA scores are shown at each corner. The number of loops used for generating APA plots is also provided beside cell type names. (TIFF) [file pcbi.1012136.s009.tiff]

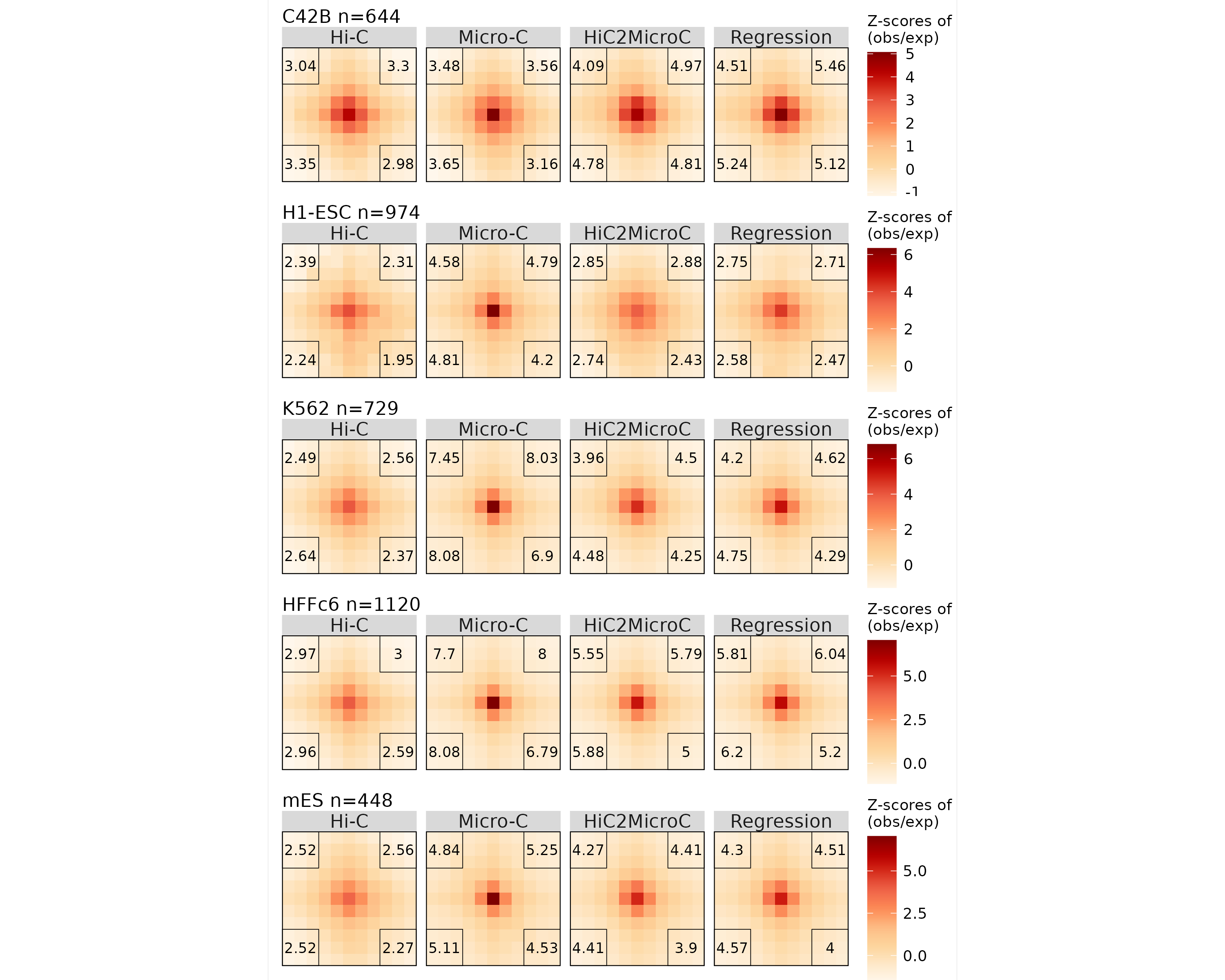

Supplement: S7 Fig — The APA scores are shown at each corner. The number of loops used for generating APA plots is also provided beside cell type names. (TIFF) [file pcbi.1012136.s010.tiff]

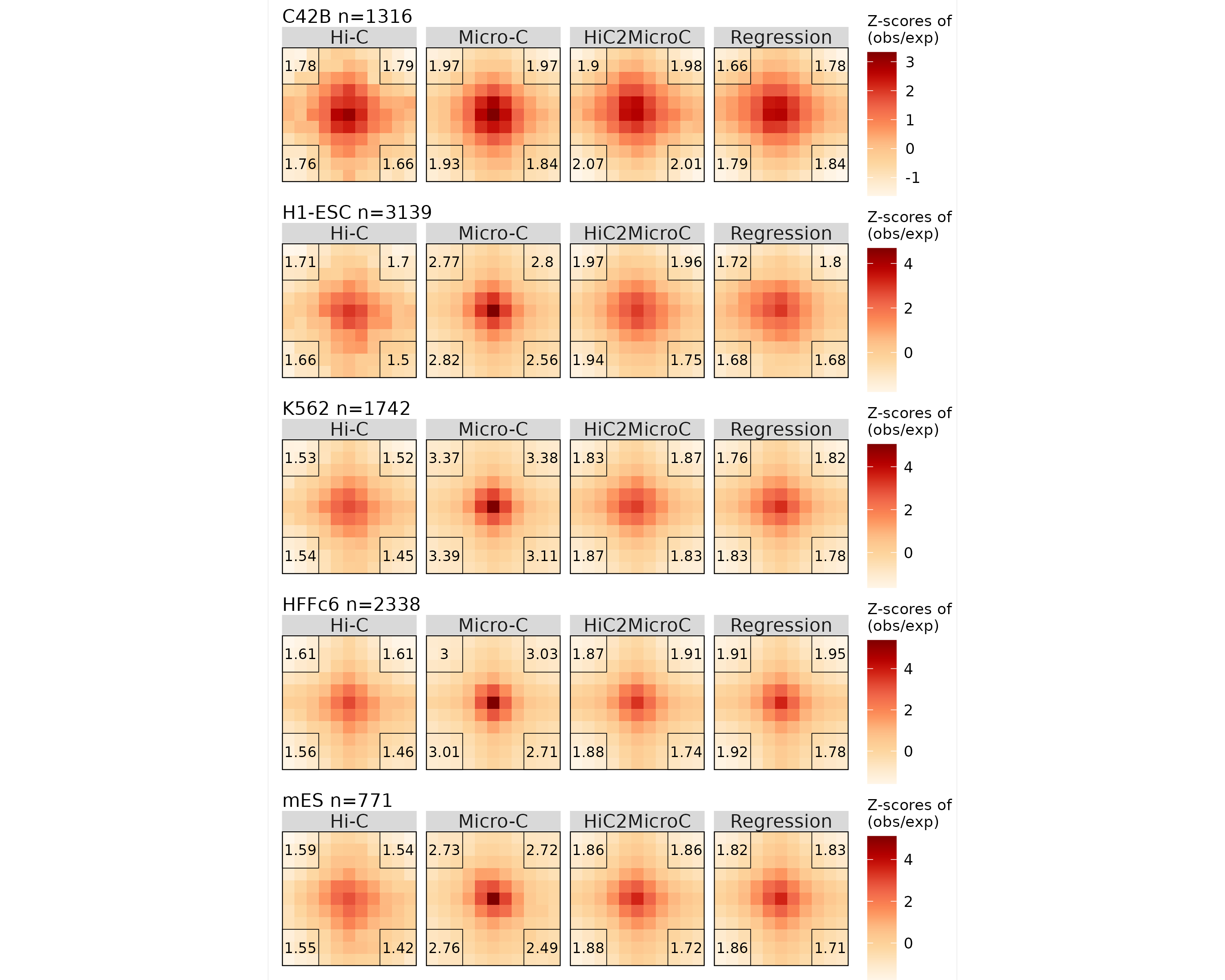

Supplement: S8 Fig — The APA scores are shown at each corner. The number of loops used for generating APA plots is also provided beside cell type names. (TIFF) [file pcbi.1012136.s011.tiff]

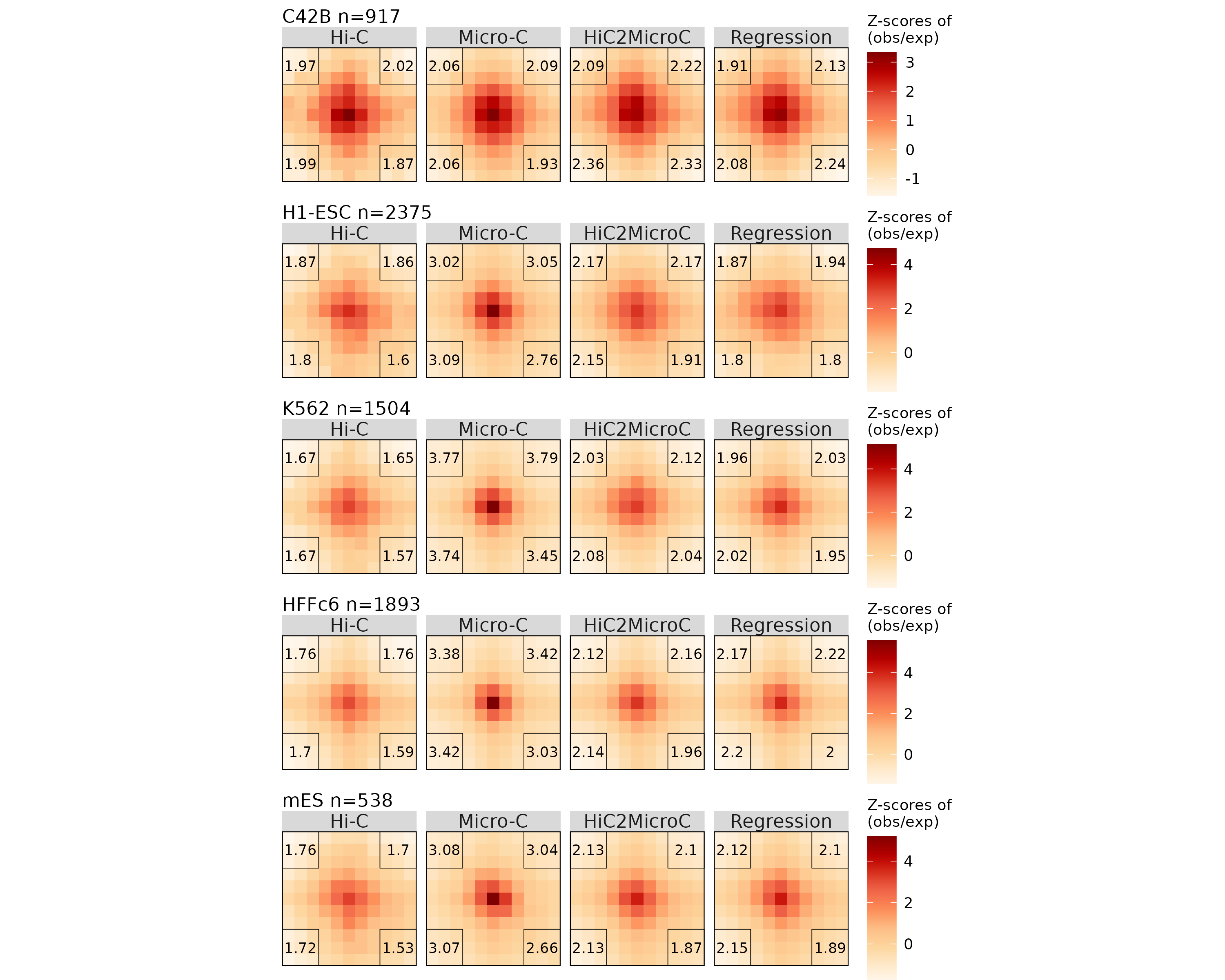

Supplement: S9 Fig — The APA scores are shown at each corner. The number of loops used for generating APA plots is also provided beside cell type names. (TIFF) [file pcbi.1012136.s012.tiff]

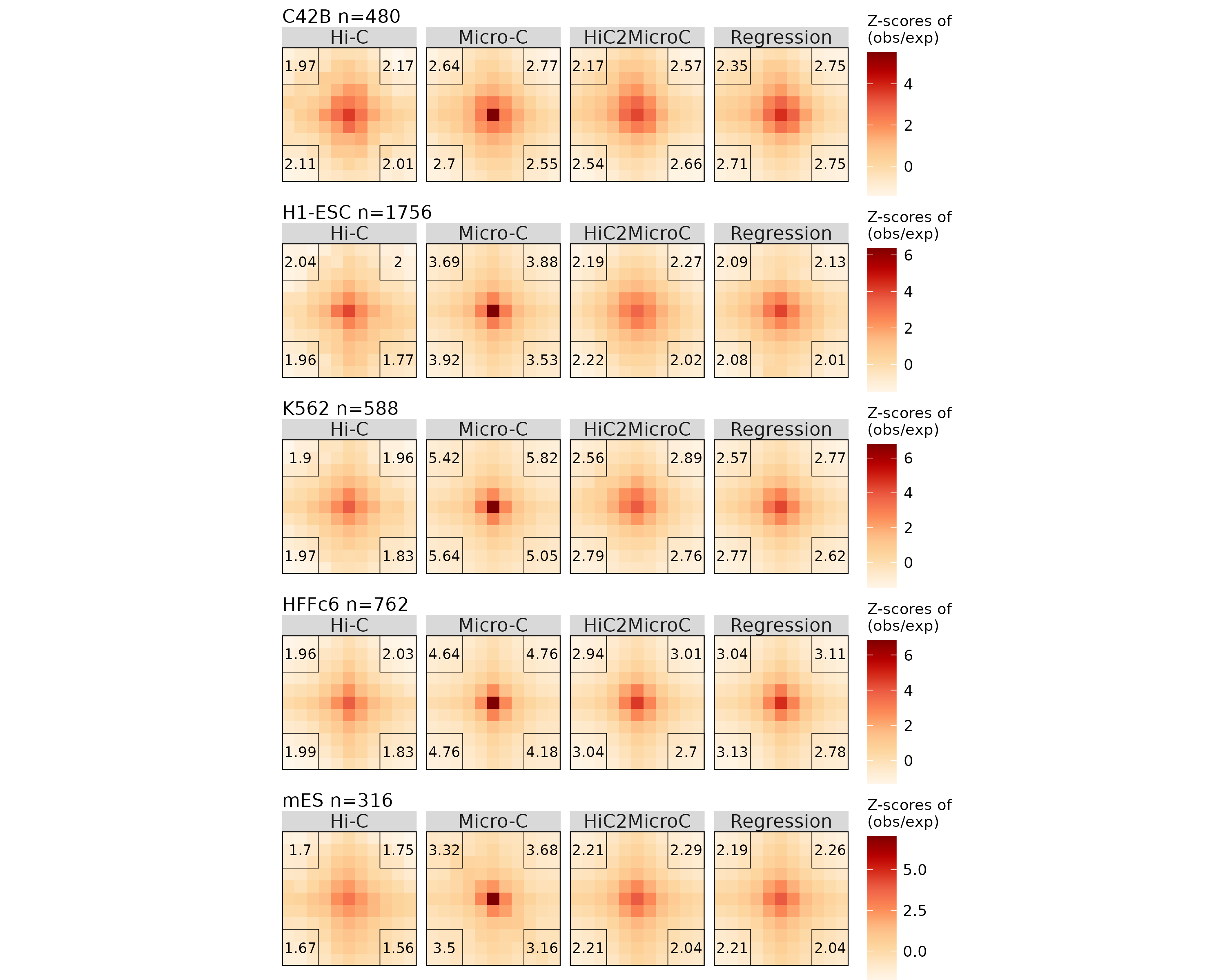

Supplement: S10 Fig — The APA scores are shown at each corner. The number of loops used for generating APA plots is also provided beside cell type names. (TIFF) [file pcbi.1012136.s013.tiff]

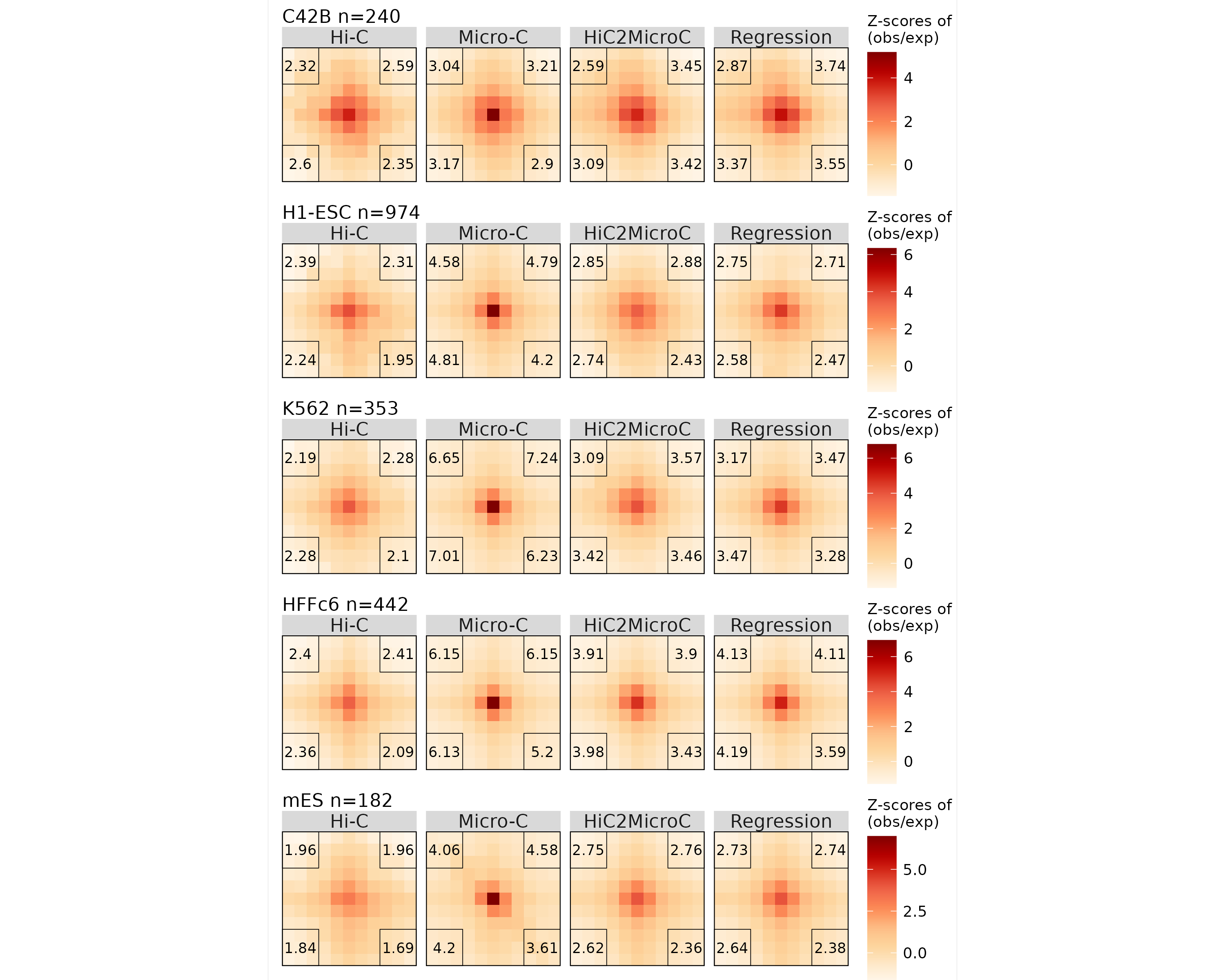

Supplement: S11 Fig — The APA scores are shown at each corner. The number of loops used for generating APA plots is also provided beside cell type names. (TIFF) [file pcbi.1012136.s014.tiff]

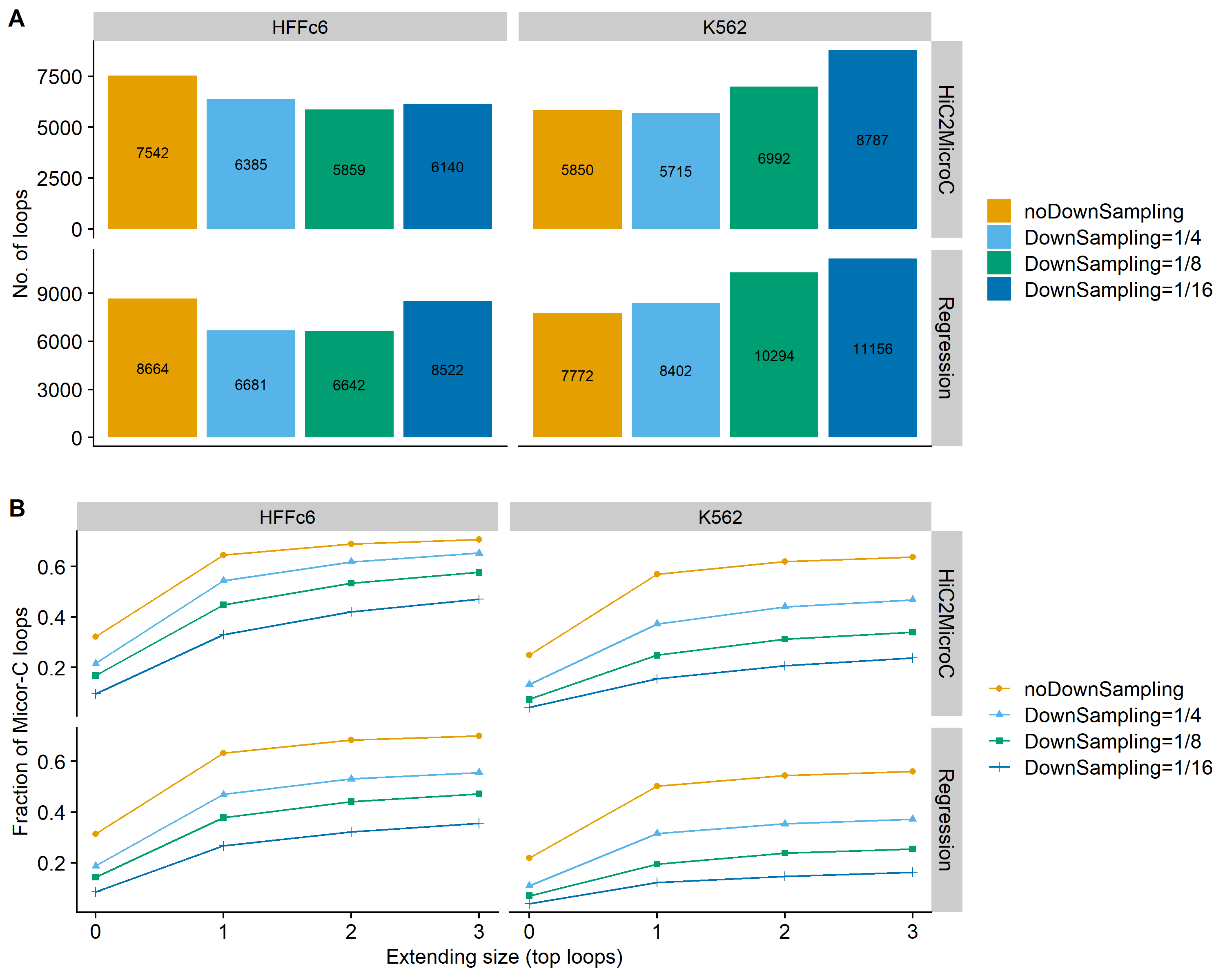

Supplement: S12 Fig — (A) The number of loops detected by Mustache (FDR = 0.05) with four types of Hi-C as input (original and three down-sampling datasets) in HFFc6 and K562. (B) The fraction of recovering Micro-C loops for top Mustache-detected loops from HiC2MicroC-predicted Micro-C with four types of Hi-C as input. (TIFF) [file pcbi.1012136.s015.tiff]

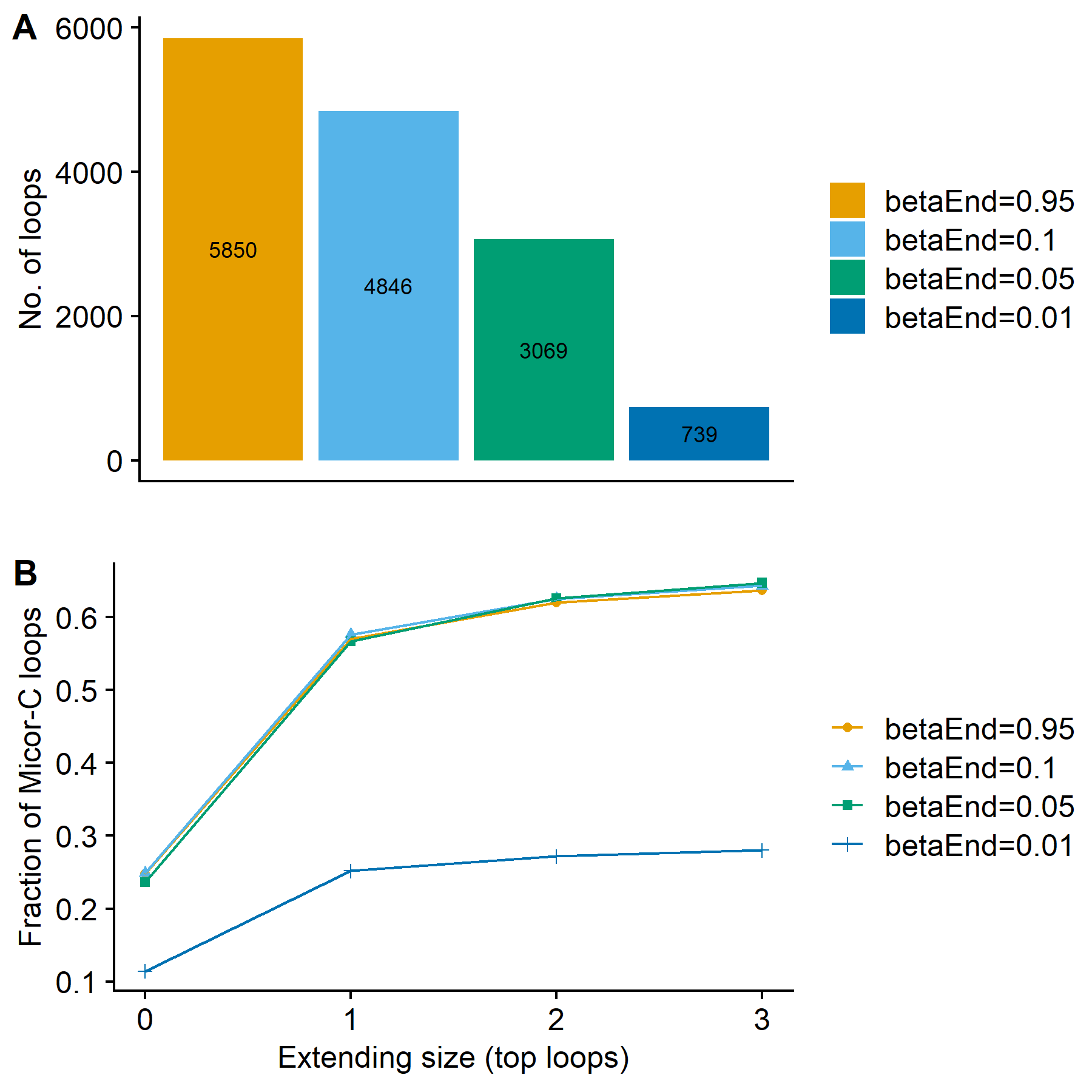

Supplement: S13 Fig — (A) The number of loops detected by Mustache (FDR = 0.05) in K562 from HiC2McroC-predicted Micro-C with four βT values (0.95, 0.1, 0.05, and 0.01) to determine noise level. (B) the Fraction of recovering Micro-C loops. (TIFF) [file pcbi.1012136.s016.tiff]

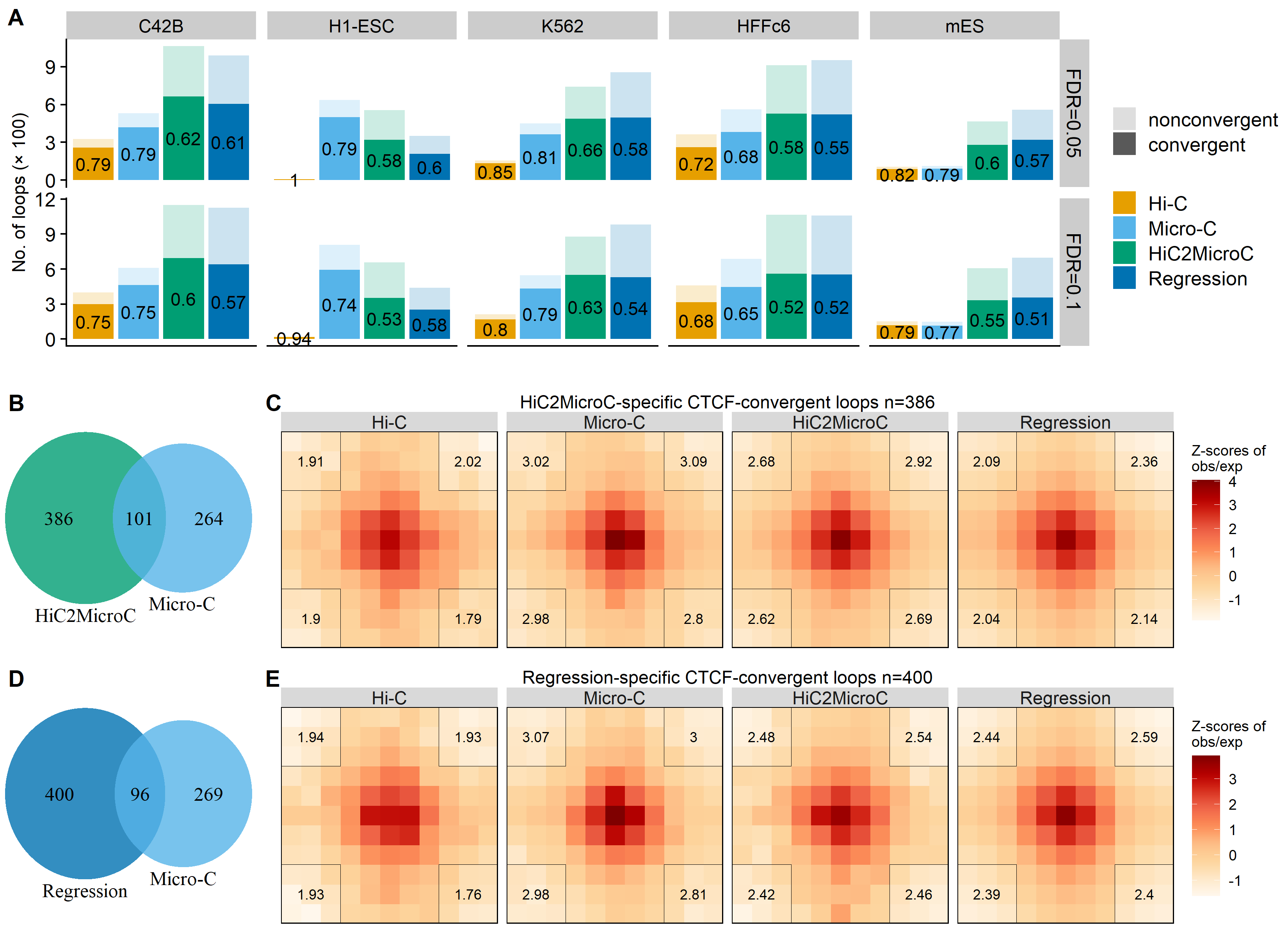

Supplement: S14 Fig — (A) The fraction of all Mustache-detected loops that have convergent CTCT binding sites. (B) The agreement of convergent loops between Micro-C and HiC2MicroC. (C) APA plots of HiC2MicroC-specific, CTCF-convergent loops (FDR = 0.05) on the four types of contact matrices. (D) The agreement of convergent loops between Micro-C and regression. (E) APA plots of regression-specific, CTCF-convergent loops (FDR = 0.05) on the four types of contact matrices. (TIFF) [file pcbi.1012136.s017.tiff]

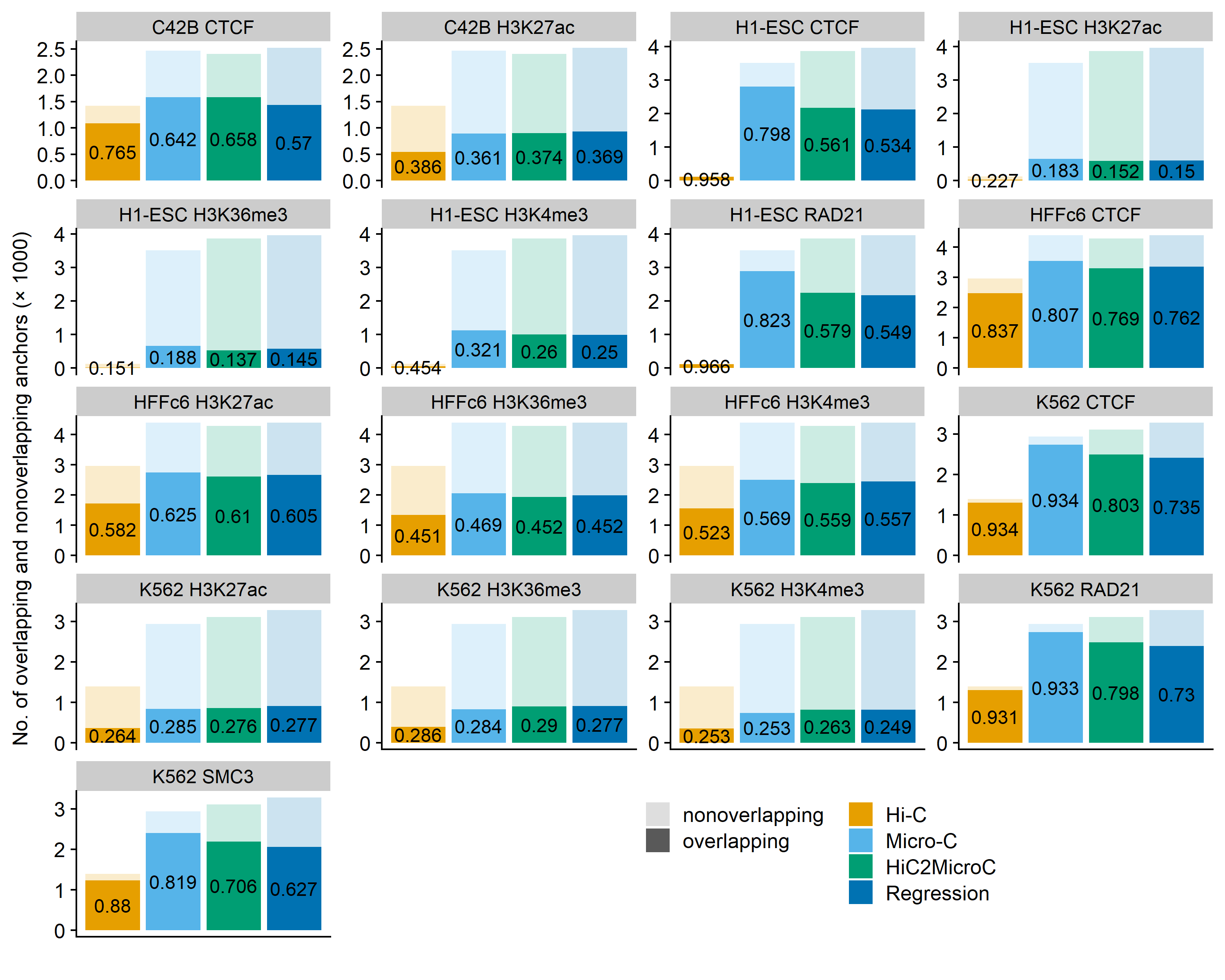

Supplement: S15 Fig — (TIFF) [file pcbi.1012136.s018.tiff]

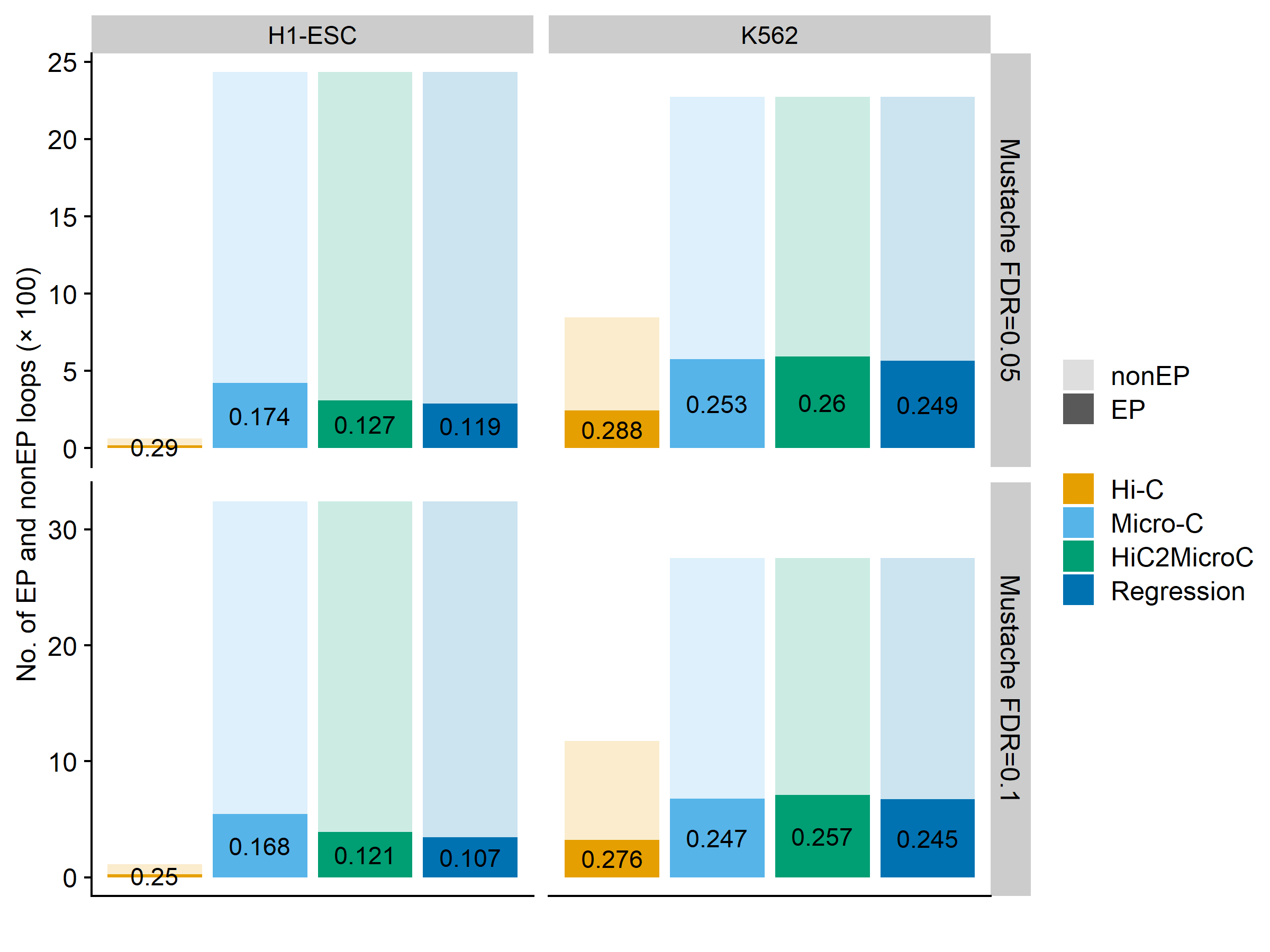

Supplement: S16 Fig — The cell-specific chromatin state annotation is used to demarcate promoter and enhancer regions. (TIFF) [file pcbi.1012136.s019.tiff]

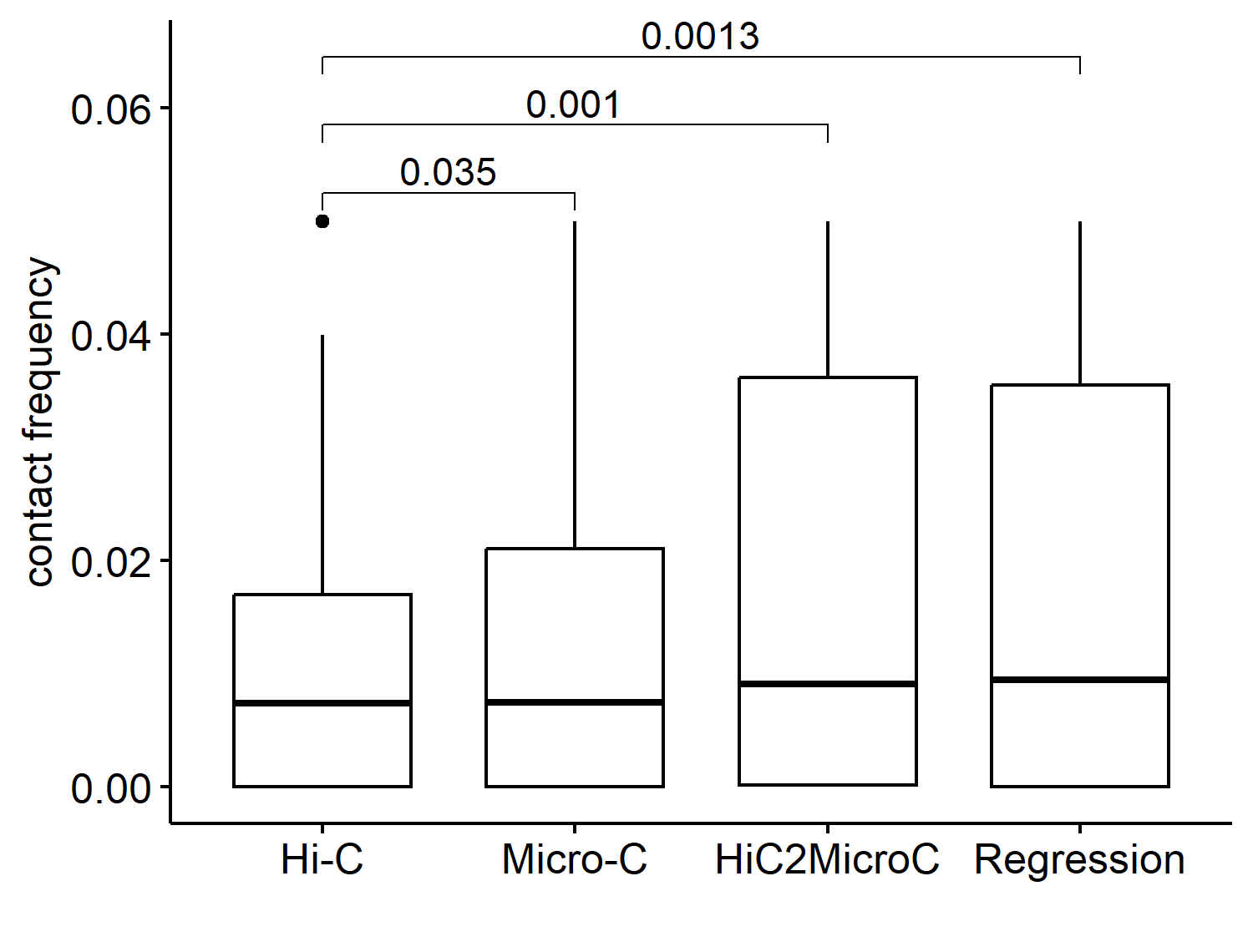

Supplement: S17 Fig — P-values are computed with the student’s t-test. (TIFF) [file pcbi.1012136.s020.tiff]

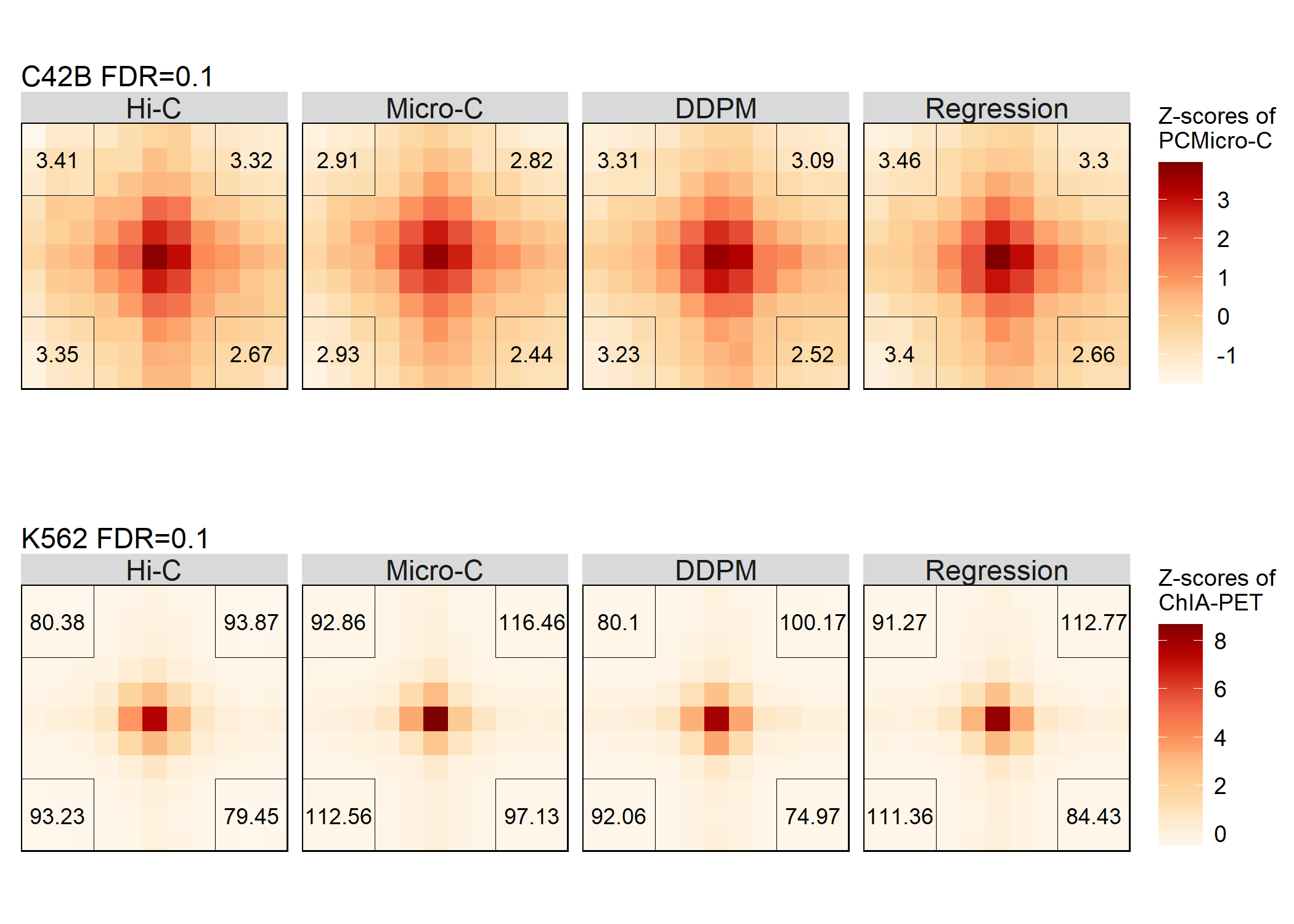

Supplement: S18 Fig — Bottom: APA plots of Hi-C and Micro-C loops (FDR = 0.1) on CTCF ChIA-PET contact matrices in K562. (TIFF) [file pcbi.1012136.s021.tiff]

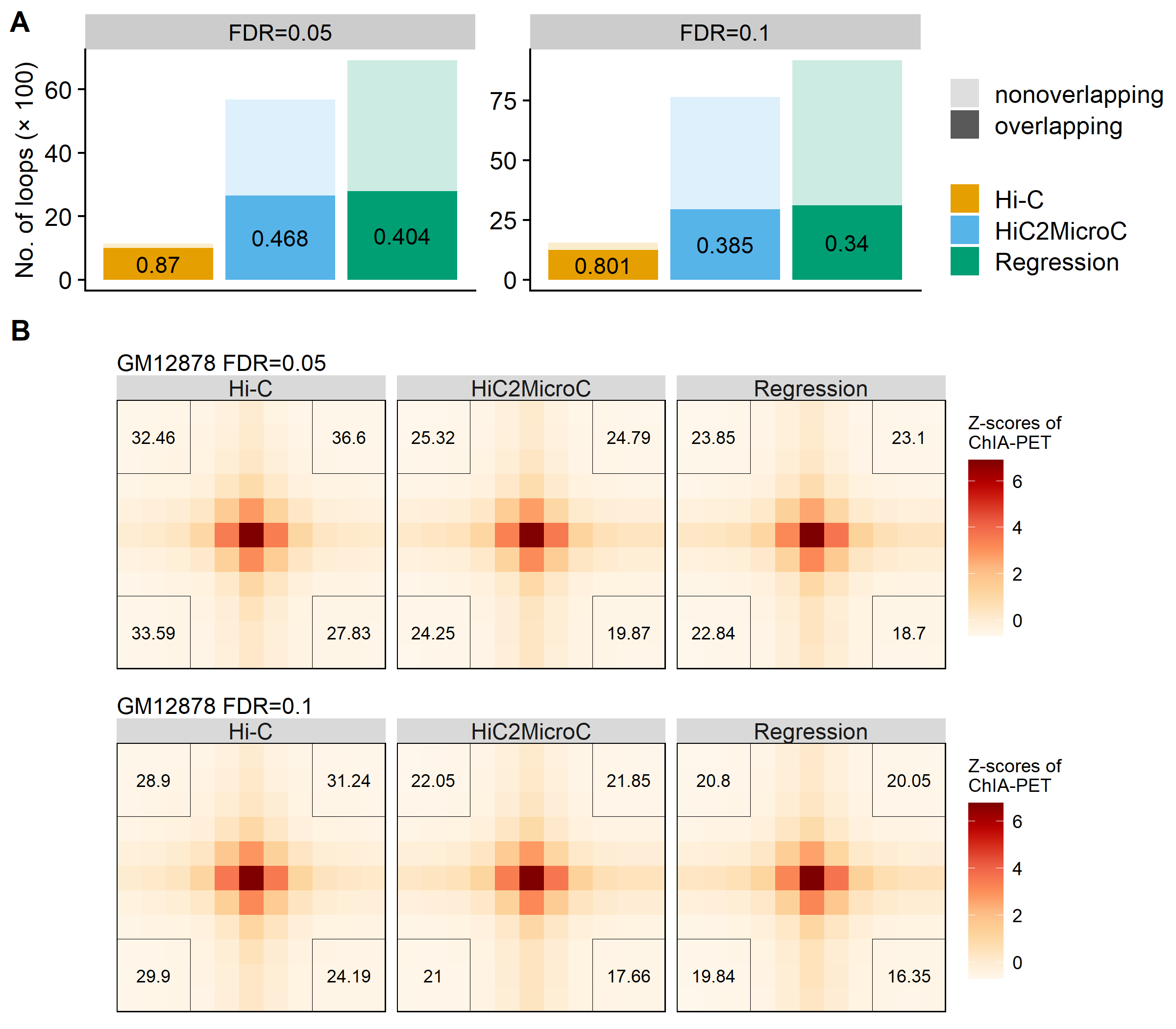

Supplement: S19 Fig — (A) Recovering CTCF ChIA-PET loops by Mustache-detected loops from Hi-C, HiC2MicroC, and regression. (B) APA plots of Hi-C and predicted-Micro-C loops within genomic distance [100kb-2Mb] on CTCF ChIA-PET contact matrices in GM12878. (TIFF) [file pcbi.1012136.s022.tiff]

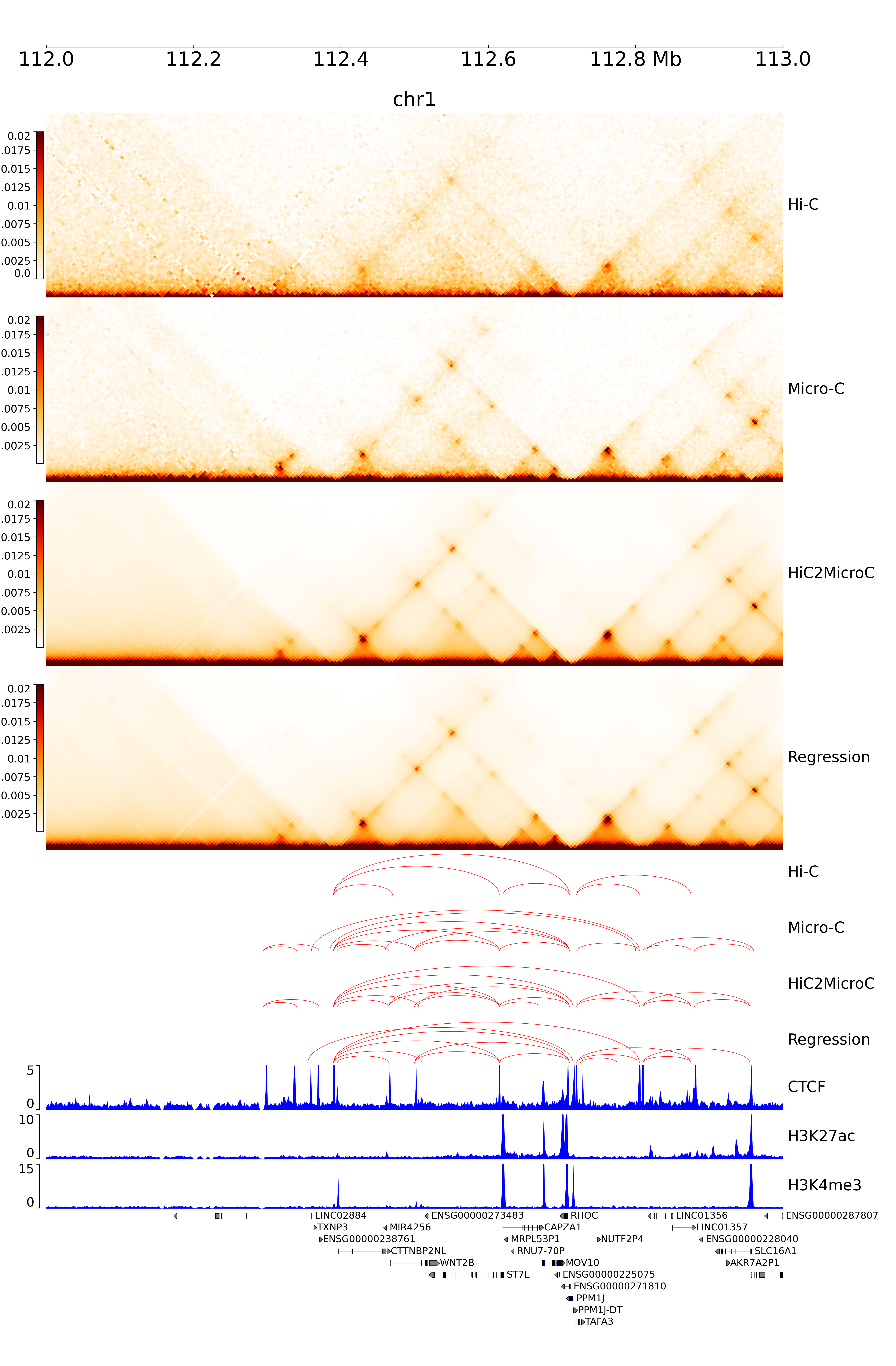

Supplement: S20 Fig — (TIFF) [file pcbi.1012136.s023.tiff]

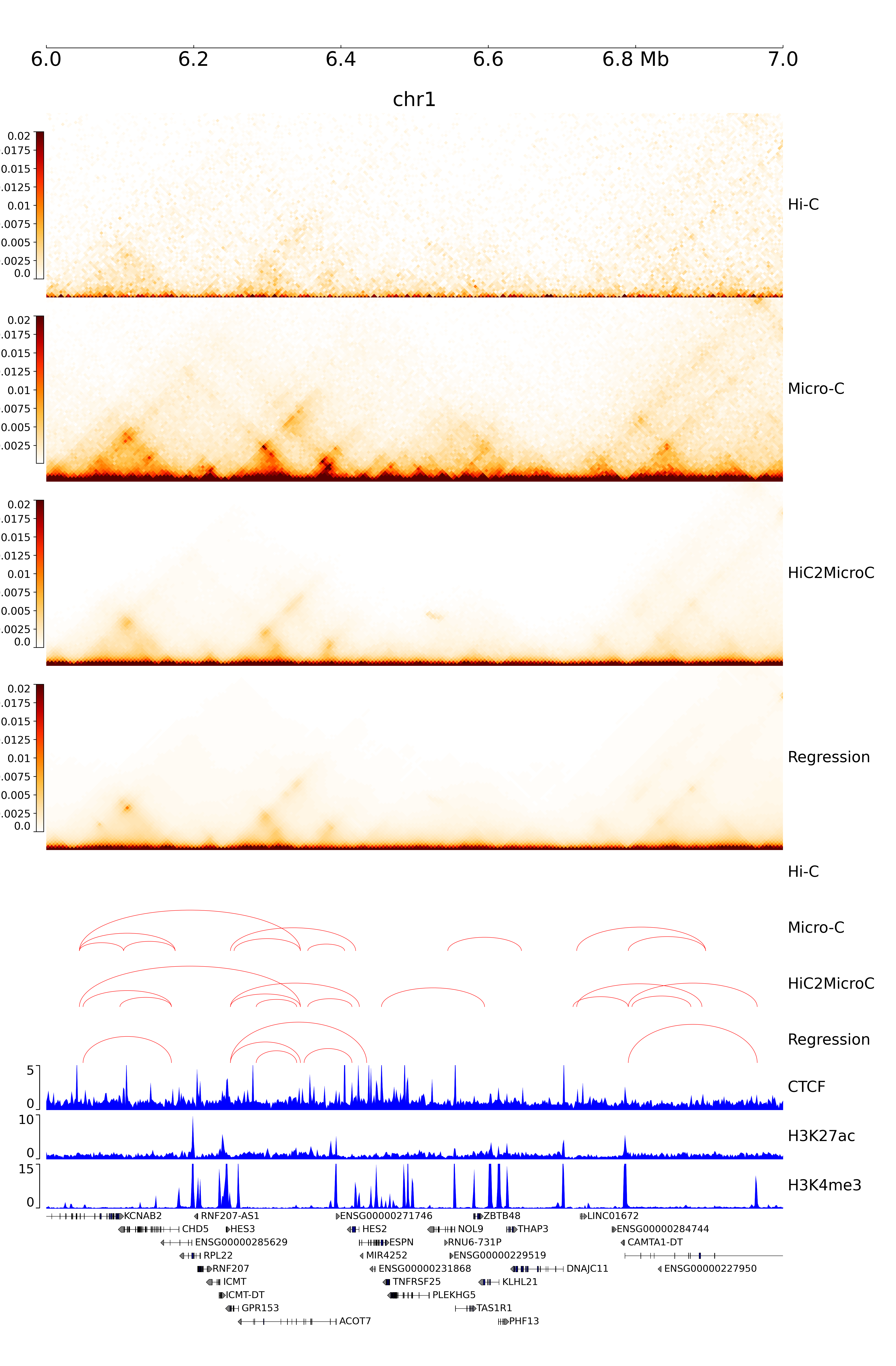

Supplement: S21 Fig — (TIFF) [file pcbi.1012136.s024.tiff]

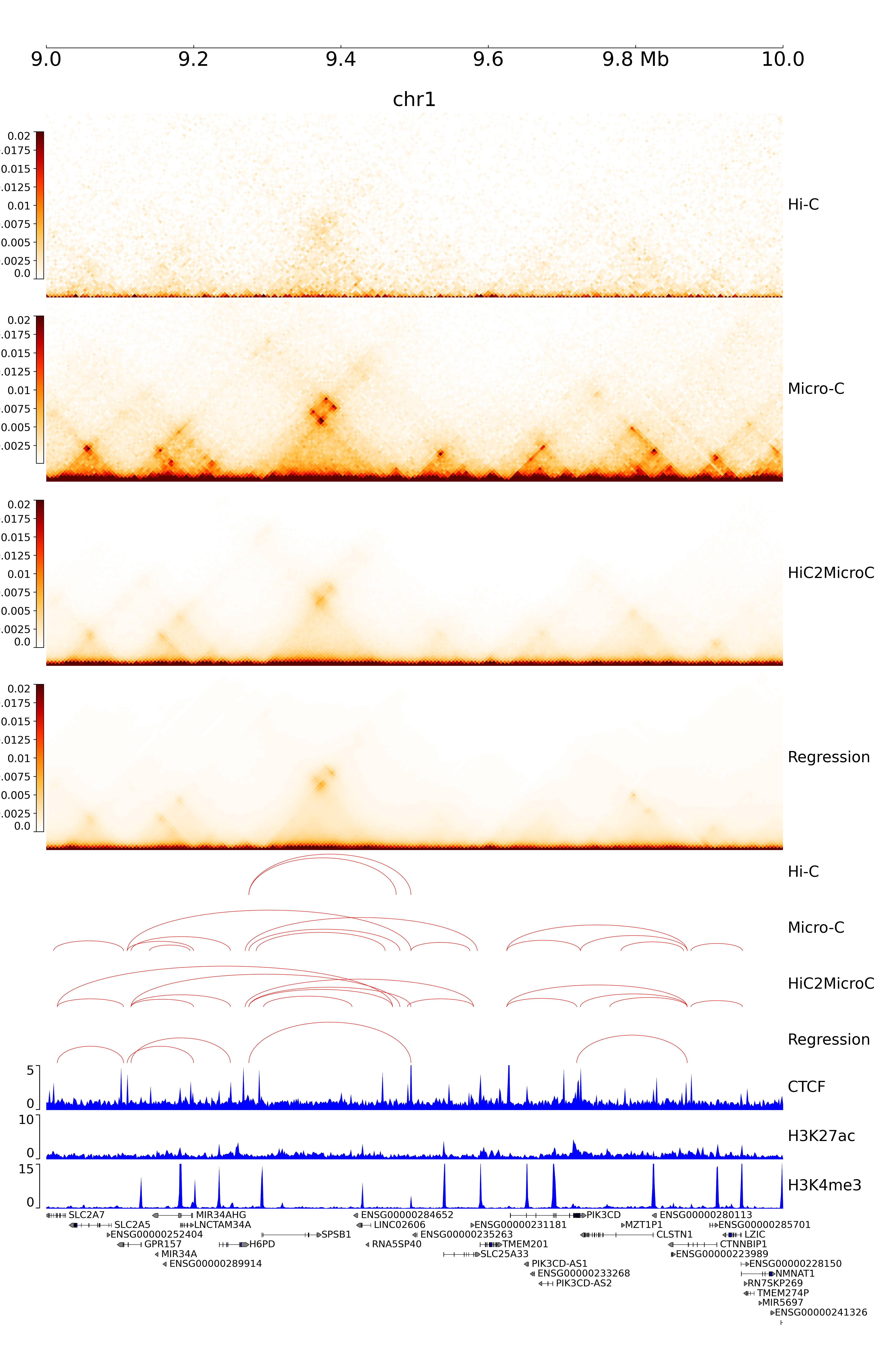

Supplement: S22 Fig — (TIFF) [file pcbi.1012136.s025.tiff]

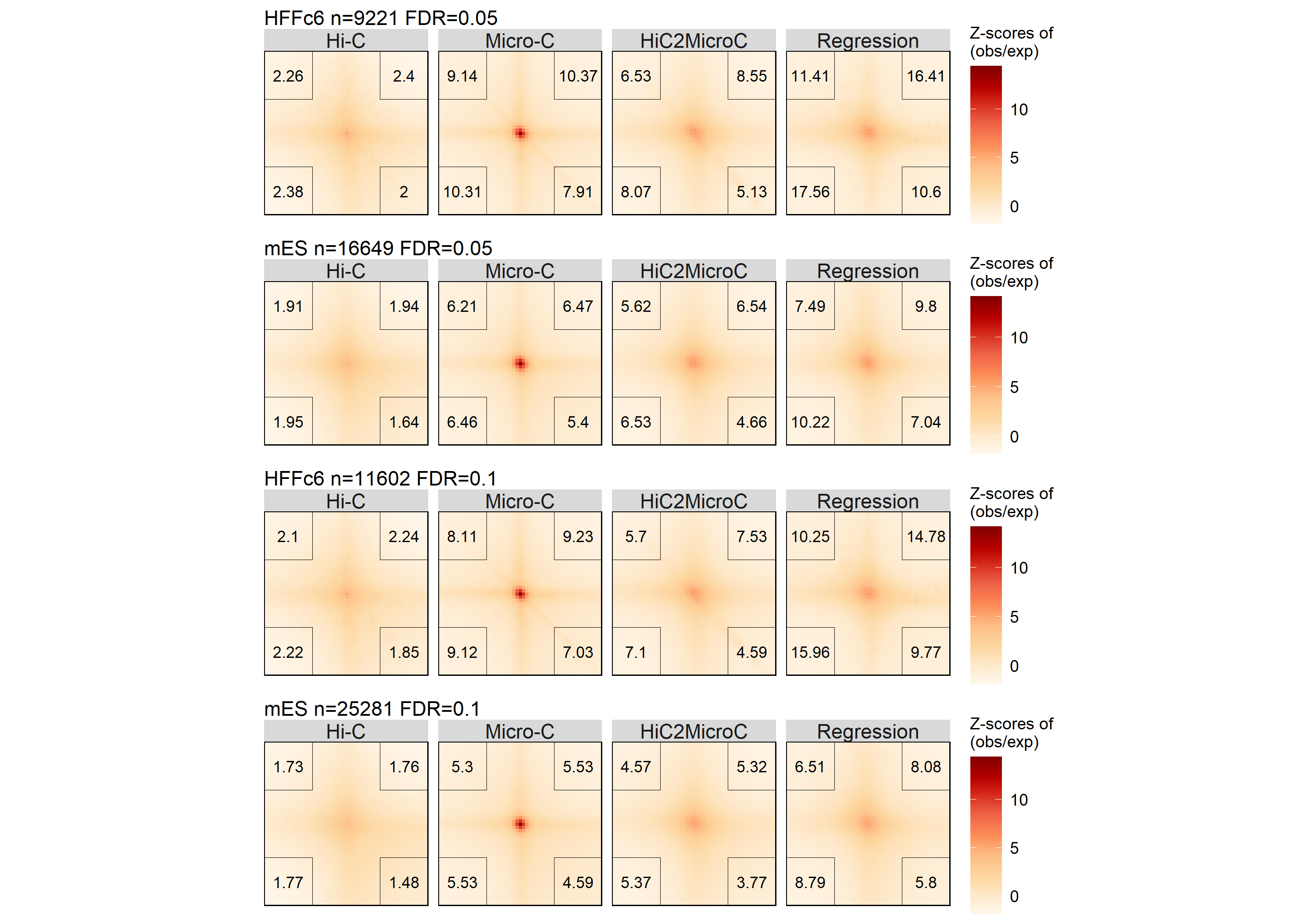

Supplement: S23 Fig — All loops are detected on 1-kb contact matrices. (TIFF) [file pcbi.1012136.s026.tiff]

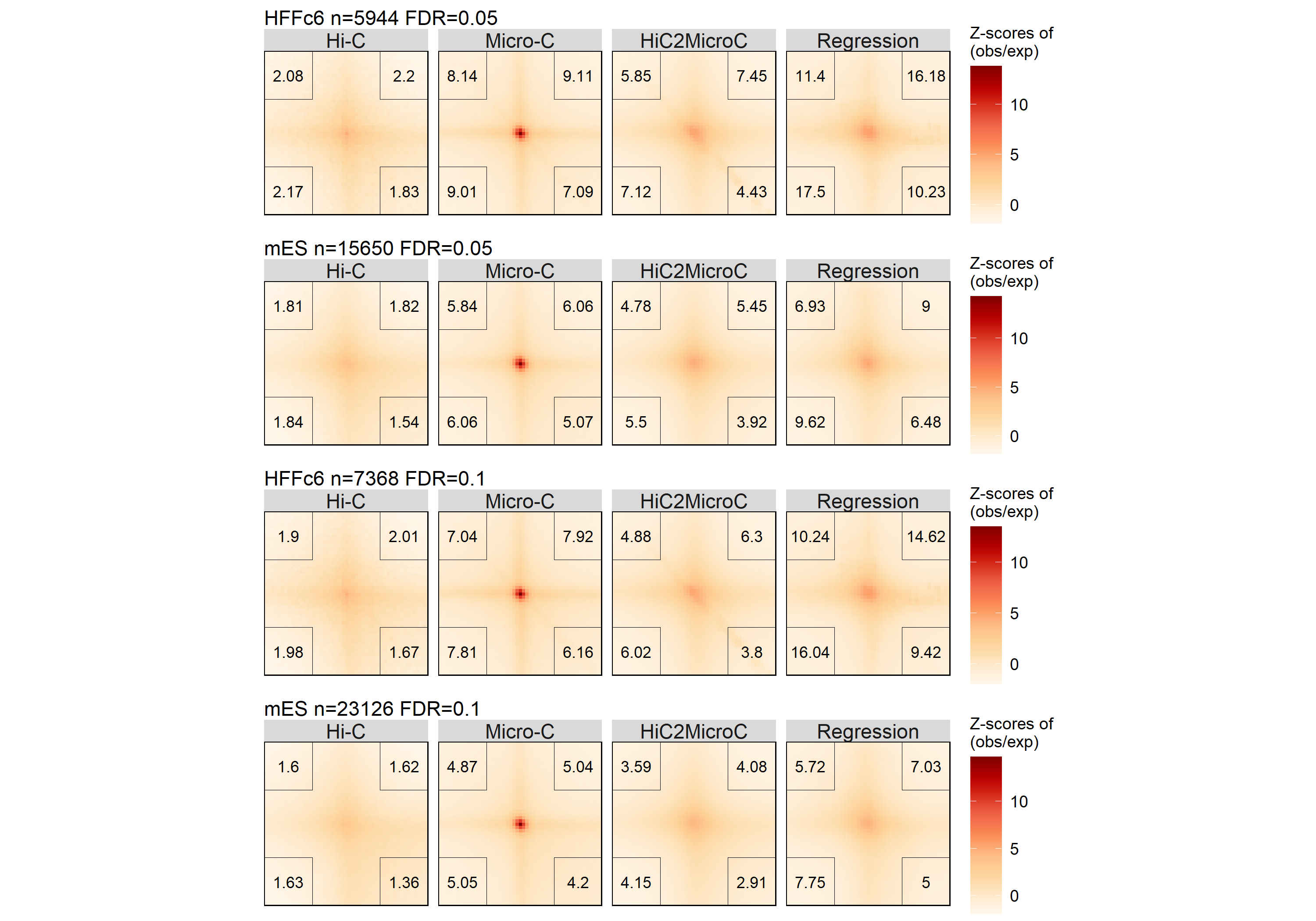

Supplement: S24 Fig — Specific loops are generated with the extending size set to four by filtering out loops that are found in Hi-C loops. All loops are detected on 1-kb contact matrices. (TIFF) [file pcbi.1012136.s027.tiff]
